# Ubiquitination of Rheb governs growth factor-induced mTORC1 activation

**DOI:** 10.1038/s41422-018-0120-9

**Published:** 2018-12-04

**Authors:** Lu Deng, Lei Chen, Linlin Zhao, Yan Xu, Xiaoping Peng, Xinbo Wang, Lin Ding, Jiali Jin, Hongqi Teng, Yanming Wang, Weijuan Pan, Fei Yu, Lujian Liao, Li Li, Xin Ge, Ping Wang

**Affiliations:** 1 Tongji Unviersity Cancer Center, Shanghai Tenth People’s Hospital, School of Medicine, School of Life Sciences and Technolog, Tongji University, 200092 Shanghai, China; 2 Institute of Biophysics, Chinese Academy of Sciences, 100101 Beijing, China; 3 College of Life Sciences, University of Chinese Academy of Sciences, 100049 Beijing, China; 4 Shanghai Key Laboratory of Regulatory Biology, Institute of Biomedical Sciences and School of Life Sciences, East China Normal University, 200241 Shanghai, China; 5 Institute of Aging Research, Hangzhou Normal University, 311121 Hangzhou, China; 6 Department of Clinical Medicine, Shanghai Tenth People’s Hospital, Tongji University School of Medicine, 200072 Shanghai, China

**Keywords:** Ubiquitylation, Ubiquitylation

## Abstract

Mechanistic target of rapamycin mTOR complex 1 (mTORC1) plays a key role in the integration of various environmental signals to regulate cell growth and metabolism. mTORC1 is recruited to the lysosome where it is activated by its interaction with GTP-bound Rheb GTPase. However, the regulatory mechanism of Rheb activity remains largely unknown. Here, we show that ubiquitination governs the nucleotide-bound status of Rheb. Lysosome-anchored E3 ligase RNF152 catalyzes Rheb ubiquitination and promotes its binding to the TSC complex. EGF enhances the deubiquitination of Rheb through AKT-dependent USP4 phosphorylation, leading to the release of Rheb from the TSC complex. Functionally, ubiquitination of Rheb is linked to mTORC1-mediated signaling and  consequently regulates tumor growth. Thus, we propose a mechanistic model whereby Rheb–mediated mTORC1 activation is dictated by a dynamic opposing act between Rheb ubiquitination and deubiquitination that are catalyzed by RNF152 and USP4 respectively.

## Introduction

The mechanistic target of rapamycin (mTOR) is a conserved serine/threonine protein kinase in all eukaryotes that incorporates various intracellular and extracellular signals including growth factors, nutrients, cellular energy, and cellular stress, and regulates cell metabolism, growth, proliferation, and survival^1–3^. mTOR is a core component of two distinct protein-signaling complexes: the rapamycin-sensitive mTOR complex 1 (mTORC1) and the rapamycin-resistant mTOR complex 2 (mTORC2). The mTORC1 complex, consisting of mTOR, mLST8, and Raptor, works as a downstream node of both Raf-MEK-ERK and PI3K-PDK1-AKT signaling pathways^4,5^. The activated mTORC1 phosphorylates various substrates including S6K, 4EBP1, ULK1, and TFEB, and regulates cell growth, autophagy, and cell metabolism. The mTORC2 complex consists of mTOR, mLST8, Rictor, and mSin1, which induces cell proliferation and survival through phosphorylation of the AGC kinase family members such as AKT and SGK^4,6^. Deregulated mTOR signaling is intimately correlated to various diseases including cancers, metabolic diseases and developmental disorders^1,4,7,8^. Therefore, mTOR is tightly controlled at multiple levels under normal conditions.

In response to a variety of environmental signals, mTORC1 is activated at lysosome through two Ras-related small G proteins, Rheb- and Rag-GTPase. Multiple regulators have been identified to regulate the activation of Rag-GTPases, such as Ragulator complex, GATOR1/2, CASTOR1, and Sestrin2^1^. Ragulator complex functions as the GEF (Nucleotide exchange factor) for RagA^9^ while GATOR1 is identified as a GAP (GTPase-activating protein) for Rag^10^. Senstrin2 mediates mTORC1 activity by acting as a GDI (guanine nucleotide dissociation inhibitor) for Rag^11^ or a protein partener with GATOR2^12,13^. In contrast to Rag GTPase, the regulation mechanism of Rheb is less understood. TSC complex, consisting of three core subunits (TSC1, TSC2, and TBC1D7), is identified as a major upstream regulator of Rheb. This complex negatively regulates mTORC1 activity by converting Rheb from its active form (GTP-bound Rheb) to the inactive form (GDP-bound Rheb)^7,14–16^. The GAP activity of TSC2 on Rheb is regulated by extracellular signals through the phosphorylation of TSC2 by AKT, AMPK, GSK3, ERK, SGK, or RSK^17–19^. However, whether TSC complex has any other function rather than a GAP of Rheb is still unclear.

Ubiquitination is a reversible posttranslational modification that is catalyzed by an enzymatic cascade including ubiquitin-activating enzyme (E1), ubiquitin-conjugating enzyme (E2), and ubiquitin ligase (E3), which can be reversed by a family of enzyme named as deubiquitinases^20^. Ubiquitination is categorized into two major types termed mono-ubiquitination and polyubiquitination. Both mono-ubiquitination and polyubiquitination are involved in a wide variety of cellular functions^20–24^. Recent studies indicate that K63-linked polyubiquitination of RagA mediated by E3 ligases RNF152 and SKP2 strongly inhibits mTORC1 activation^25,26^. Meanwhile, TRAF6-mediated K63-plolyubiquitination of mTOR is essential for mTORC1 activation^27^. In parallel, TRAF2 and OTU7b  govern mTORC2 activation by targeting GβL for K63-linked polyubiquitination^28^. However, whether ubiquitination of Rheb manipulates mTORC1 activation is unclear.

In current study, we found that TSC2 inactivates Rheb by promoting Rheb ubiquitination. Lysosomal E3 ligase RNF152 targets Rheb for ubiquitination at K8 site and sequesters Rheb in its inactive form (Rheb-GDP), leading to the abolishment of GTP reloading followed by mTORC1 inactivation in an EGF-sensitive manner. Upon growth factor stimulation, deubiquitinase USP4 was phosphorylated by AKT, resulting in the release of the inhibitory TSC complex from Rheb, which is essential for the activation of both Rheb and mTORC1. Our data indicate that RNF152 and USP4 constitute an intricate regulatory network that controls the transformation between Rheb-GDP (Rheb inactivation state) and Rheb-GTP (Rheb activation state), thereby critically affecting mTORC1 activation, autophagy, cell proliferation, and tumorigenesis. Moreover, genetic deletion of USP4 or treatment with USP4 inhibitor, Vialinin A,  inhibits colorectal tumor growth, implicating the potential clinical applications of USP4 inhibitors for future cancer  therapy. Collectively, our study reveals an in facto regulatory mechanism by which the post-translational modification of Rheb determines mTORC1 activation and consequent tumorigenesis.

## Results

### The TSC complex and EGF signaling regulate the ubiquitination of Rheb

**Fig. 1:**
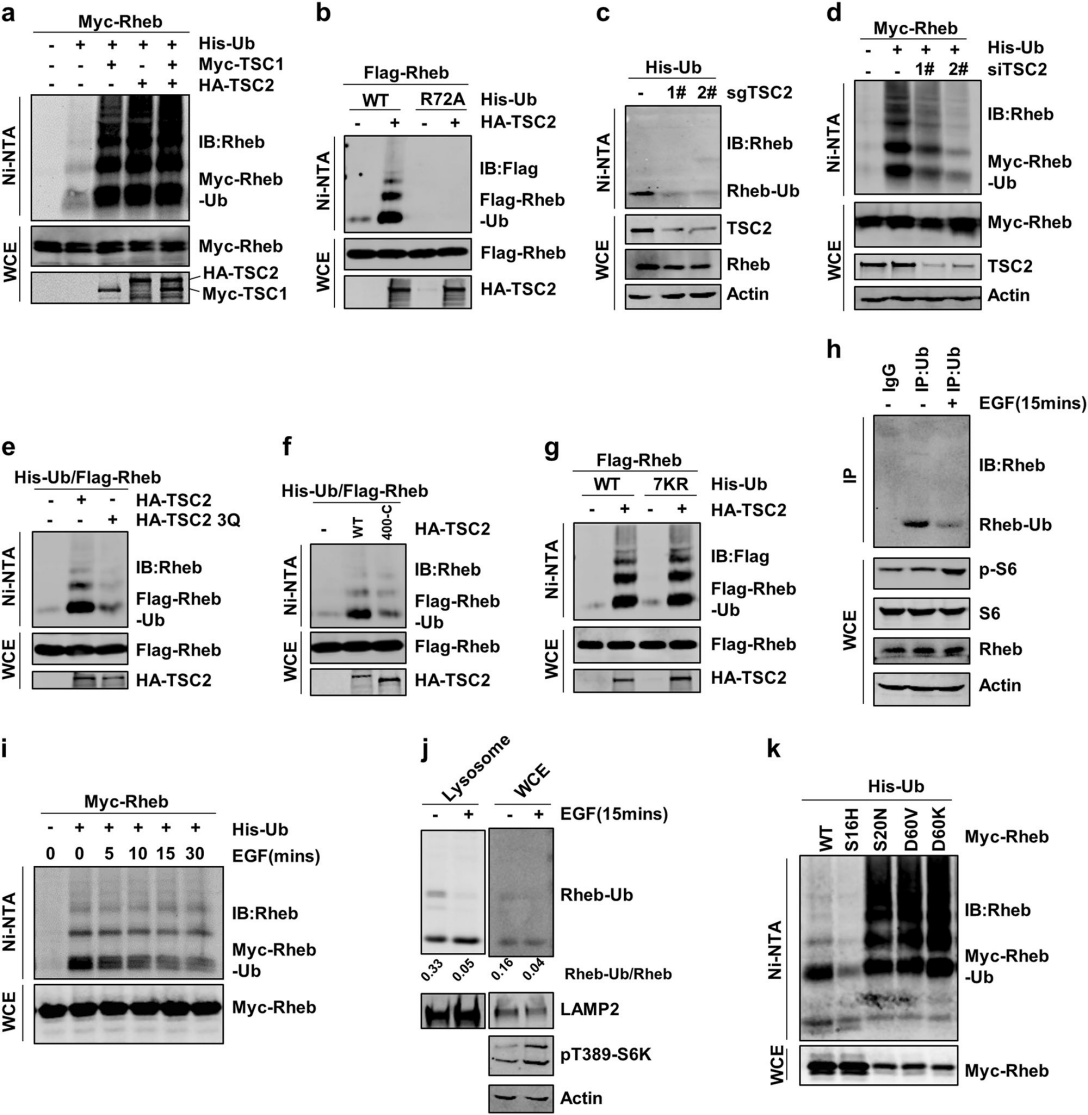
TSC complex and EGF signaling promote the ubiquitination of Rheb. **a** TSC1 and TSC2 promoted Rheb ubiquitination in HEK293T cells. The ubiquitinated proteins were purified under the denature condition via Ni-NTA agarose beads and were analyzed by Western Blotting. **b** Co-transfection of HA-TSC2, Myc-Rheb, Ub-WT, or Ub-R72A mutants to detect Rheb ubiquitination, Rheb ubiquitination was analyzed as in (**a**). **c**, **d** Rheb ubiquitination was detected in *TSC2* knockdown cells. **e**, **f** Ubiquitination of Rheb was detected by co-transfection of TSC2-WT, TSC2-3Q mutant (**e**) or TSC2(400-C) mutant (**f**) in HEK293T cells. **g** The linkage-specific ubiquitination of Rheb was examined in HEK293T cells. **h**, **i** Ubiquitination of endogenous (**h**) and exogenous (**i**) Rheb was detected in HEK293T cells with the treatment of EGF. **j** Rheb ubiquitination level was detected in lysosome samples enriched from cells with or without EGF treatment. **k** Ubiquitination of WT, active (S16H) and inactive (D60V, D60K, S20N)) forms of Rheb was analyzed via Ni-NTA

As a key negative regulator of mTORC1 signaling pathway, the TSC complex was reported to mainly function as a GAP to inactivate Rheb GTPase^17^. Surprisingly, we found that the expression of TSC1, TSC2, or TBC1D7 promoted Rheb ubiquitination in HEK293T cells (Fig. 1a, Supplementary information, Fig. S1a, b). TSC2-induced Rheb ubiquitination was confirmed using ubiquitin mutant (R72A) as a negative control, which could not be activated by ubiquitin-activating enzyme E1^29^ (Fig. 1b and Supplementary information, Fig. S1c). Our data from a sequential purification approach under the denature conditions (Supplementary information, Fig. S1d, e) and the ubiquitination assay using *Rheb* knockdown cells (Supplementary information, Fig. S1f) further demonstrated that ubiquitination of Rheb is dependent on TSC2. In addition, depletion of TSC1 or TSC2 using their specific siRNAs or sgRNAs reduced the ubiquitination level of Rheb (Fig. 1c, d and Supplementary information, Fig. S1g), indicating that endogenous TSC complex is responsible for Rheb ubiquitination.

Given that TSC2 is a GAP for Rheb, we investigated whether the GAP activity of TSC2 is involved in Rheb ubiquitination. Our data showed that the GAP activity-deficient mutant, TSC2–3Q (refer to K1595Q, K1596Q, R1597Q)^30^, lost the ability to promote Rheb ubiquitination (Fig. 1e) and failed to inhibit mTORC1 activation (Supplementary information, Fig. S1h). Moreover, the TSC2 mutant lacking its N-terminal residues from 1 to 399 (400-C) which is required for its interaction with TSC1 and essential for its GAP activity^30^, lost the ability to induce Rheb ubiquitination (Fig. 1f and Supplementary information, Fig. S1i). These data together indicate that the GAP activity of TSC complex is required for Rheb ubiquitination.

To determine which type of linkage-specific ubiquitin chain was anchored on Rheb, we co-expressed Rheb with different lysine-mutated ubiquitin mutants in HEK293T cells and found that all the expressed ubiquitin mutants, including lysine free mutant (Ub-7KR), had no effect on TSC2-induced Rheb ubiquitination (Fig. 1g and Supplementary information, Fig. S1j). Because the ubiquitin mutants contain His-tag at their N-terminus, the linear-ubiquitination was very unlikely. Thus, these data suggest that TSC2 might mainly promote the mono-ubiquitination of Rheb.

It has been well-established that TSC activity on Rheb is controlled by growth factors^1^. Our data showed that EGF treatment reduced ubiquitination of both endogenous and exogenous Rheb (Fig. 1h, i). In contrast, the amino acids had little effect on Rheb ubiquitination (Supplementary information, Fig. S1k).

Although Rheb is found to be localized on the endomembrane systems including lysosome, Golgi and ER, it is mainly activated on the lysosomal membrane to regulate mTORC1 activation^31,32^. We therefore examined whether Rheb is ubiquitinated on the lysosome by purifying the intracellular lysosome and found that lysosomal Rheb was indeed largely ubiquitinated upon starvation and such ubiquitination was suppressed when treated with EGF (Fig. 1j and Supplementary information, Fig. S1l).

Since TSC2 inactivates Rheb and promotes its transformation from GTP- to GDP-bound form, we examined whether Rheb ubiquitination is affected by its nucleotide-bound status. Our data showed that ubiquitination of Rheb inactive mutants, including Rheb-S20N, Rheb-D60V, and Rheb-D60K, was much stronger than that of its active mutant Rheb-S16H (Fig. 1k). These data suggest that the ubiquitination is tightly correlated to Rheb inactivation.

### RNF152 acts as a direct E3 ubiquitin ligase for Rheb

**Fig. 2:**
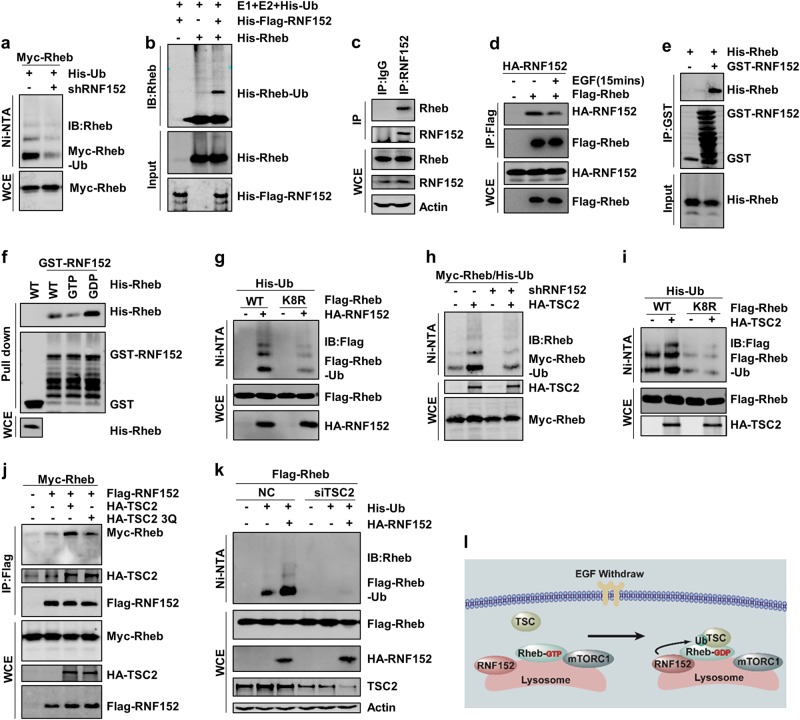
RNF152 is involved in TSC2-mediated Rheb ubiquitination. **a** Rheb ubiquitination was detected in *RNF152* knockdown cells. The knockdown efficiency of *RNF152* was detected via RT-PCR in Supplementary information, Fig. S2d. **b** RNF152 enhanced Rheb ubiquitination in vitro. **c**, **d** Endogenous(**c**) and exogenous (d) interaction between Rheb and RNF152 was examined in HEK293T cells with EGF stimulation. **e** RNF152 could specifically interact with His-tagged Rheb detected by GST pull-down assay. **f** Pull-down assay was performed to detect the interaction between GST-RNF152 and His-Rheb, and Rheb was loaded with GDP or GTP in advance. **g** Rheb-K8R displayed lower ubiquitination level than Rheb-WT when co-expressed with RNF152. **h** TSC2-induced Rheb ubiquitination decreased in *RNF152* knockdown HEK293T cells. The knockdown efficiency was detected by RT-PCR in Supplementary information, Fig. S2d. **i** Ubiquitination of Rheb-WT or Rheb-K8R mutant was detected with or without TSC2 in HEK-293T cells. **j** Co-IP assay was performed to test the interaction between Rheb and RNF152 after co-expressed with TSC2 or TSC2-3Q mutant. **k** Ubiquitination of Rheb was detected in TSC2-depleted HEK293T cell line. **l** The model of Rheb ubiquitination: TSC complex inactivates Rheb in the absence of EGF, which consequently promotes Rheb-GDP to interact with RNF152 followed by subsequent Rheb ubiquitination

Since ubiquitination is usually catalyzed by E3 ubiquitin ligase, we screened a panel of E3 ligases to identify the specific E3 ligase for Rheb ubiquitination. We found that ectopic expression of lysosome-localized RNF152 induced Rheb ubiquitination (Supplementary information, Fig. S2a, b and c). Consistently, ubiquitination of both endogenous and exogenous Rheb was reduced in *RNF152* knockdown cell line (Fig. 2a and Supplementary information, Fig. S2d, e and f). The E3 ligase dead mutant, RNF152-CS, failed to induce Rheb ubiquitination (Supplementary information, Fig. S2b, g). To further confirm that RNF152 is a direct E3 ligase for Rheb, we purified RNF152 and Rheb from *E. coli* respectively, and performed an in vitro ubiquitination assay. Our data clearly demonstrated that RNF152 targets Rheb for ubiquitination in vitro (Fig. 2b).

We next examined whether RNF152 is a binding partner of Rheb. Our data showed that both exogenous and endogenous RNF152 could interact with Rheb in co-immunoprecipitation and in vitro pull down assay (Fig. 2c–e). We also examined whether GTP/GDP loading affects the interaction between Rheb and RNF152. We found that RNF152 preferentially bound to GDP-loaded Rheb (Fig. 2f), indicating that the interaction between Rheb and RNF152 is affected by the nucleotide-bound status of Rheb. The fragment from 105 to 165 amino acid residues (aa) of RNF152 was essential for its interaction with Rheb (Supplementary information, Fig. S2h). Meanwhile, deletion of RNF152 transmembrane domain abolished its binding to Rheb (Supplementary information, Fig. S2h), suggesting that the lysosome localization of RNF152 mediated by its transmembrane domain is required for its binding to Rheb. Taken together, these data indicate that RNF152 is a direct binding partner for Rheb.

Rheb mediates nucleotide binding and hydrolysis through its G1-G5 boxes^33^. To map the ubiquitination sites of Rheb promoted by RNF152, we replaced each lysine residue located outside of Rheb G1–G5 boxes^33^ with arginine residue and examined RNF152-mediated ubiquitination of these mutants. Our data showed that RNF152-induced ubiquitination of Rheb-K8R (lysine 8 was replaced with arginine), but not other mutants, was reduced (Fig. 2g and Supplementary information, Fig. S2i), suggesting that the K8 residue of Rheb is the major targeting site for its ubiquitination.

### RNF152 is involved in TSC2-mediated Rheb ubiquitination

We next investigated whether RNF152 is involved in TSC2-mediated Rheb ubiquitination. Our data showed that ectopical expression of RNF152 enhanced TSC2-mediated Rheb ubiquitination (Supplementary information, Fig. S2j). On the other hand, the TSC2-induced Rheb ubiquitination was reduced in RNF152-deficient cells (Fig. 2h). Moreover, ubiquitination of Rheb-K8R mutant in the presence of TSC2 was depressed as compared with that of wild-type Rheb (Rbeb-WT) (Fig. 2i). These data together indicate that TSC2-mediated Rheb ubiquitination is largely dependent on RNF152.

To investigate how TSC2-induced Rheb ubiquitination is mediated by RNF152, we examined whether TSC2 affects the interaction between RNF152 and Rheb. Our data showed that co-expression of WT-TSC2, but not the TSC2 mutant that loses GAP activity, promoted the interaction between RNF152 and Rheb (Fig. 2j). Consistently, we found that depletion of TSC2 abolished the RNF152-induced Rheb ubiquitination (Fig. 2k). These data indicate that TSC2 promotes the binding of Rheb to RNF152 in a TSC2 GAP activity-dependent manner.

We next examined whether the interaction between RNF152 and Rheb is dependent on the nucleotide-bound status of Rheb. Our data showed that the binding of RNF152 to Rheb-S20N and Rheb-D60V was much stronger than its binding to WT and the active mutant Rheb-S16H (Supplementary information, Fig. S2k). These results are consistent with our data that the interaction between RNF152 and Rheb was inhibited by EGF treatment (Fig. 2d), which induced the GTP-bound status of Rheb. Taken together, we proposed a model that either TSC complex or EGF withdraw induces the GDP-bound Rheb, thereby promoting the interaction between Rheb and RNF152 and resulting in Rheb ubiquitination (Fig. 2l).

### Deubiquitination of Rheb by USP4

**Fig. 3:**
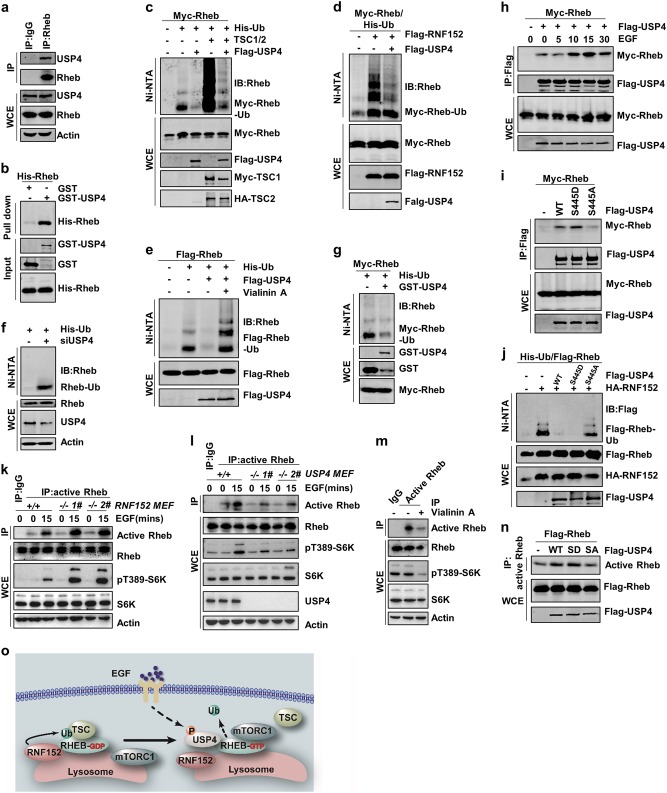
USP4 promotes Rheb activation through removing the ubiquitin from Rheb. **a** The endogenous interaction between USP4 and Rheb was analyzed via Co-IP assay. **b** The interaction between USP4 and Rheb was analyzed via pull-down assay. **c**, **d** USP4 reversed the effect of TSC2 (**c**) and RNF152 (**d**) on Rheb ubiquitination in HEK293T cells. **e** Vialinin A blocked the effects of USP4 on Rheb deubiquitination. **f** Endogenous ubiquitination of Rheb was increased in USP4-depleted HEK293T cells. **g** USP4 directly removed the ubiquitin from Rheb in vitro. **h** The interaction between Rheb and USP4 was analyzed under EGF treatment for indicated time periods. **i** The interaction between Rheb and USP4-WT, USP4-S445A, and USP4-S445D. **j** The effects of USP4-WT, USP4-S445A, and USP4-S445D on RNF152-mediated ubiquitination of Rheb in HEK293T cells. **k**, **l** The activation of Rheb was measured in RNF152 deficient (**k**) or USP4 deficient MEF cells (**l**) with EGF treatment for indicated time. RNF152-KO MEF cells were analyzed via genomic DNA PCR in Supplementary information, Fig. S3s. **m** Vialinin A reduced the activation of endogenous Rheb. **n** The activation of Rheb was measured in HEK293T cells co-expressing Flag-Rheb with USP4-WT or USP4 mutants, respectively. **o** The model of Rheb deubiquitination by USP4

Ubiquitination is a reversible process that is counteracted by deubiquitination^20,34^. To identify the deubiquitinase of Rheb, we screened a wide variety of DUBs and found that overexpression of several DUBs, including USP4, USP5, USP11, USP15, and USP22, could remove the ubiquitin chains from Rheb (Supplementary information, Fig. S2a). However, our data showed that only USP4 interacted with Rheb (Supplementary information, Fig. S2b). The interaction between USP4 and Rheb in cells was confirmed by both in vivo co-IP assay and in vitro GST pull down assay (Fig. 3a, b). Moreover, our data suggest that the DUSP domain of USP4 accounts for the interaction between Rheb and USP4 (Supplementary information, Fig. S3c, d).

We next examined whether USP4 is a deubiquitinase for Rheb. Our data showed that ectopical expression of wild-type USP4 (USP4-WT), but not the enzyme-dead mutant USP4-CS, in which cysteine 311 was replaced with arginine^35^, abrogated TSC2/RNF152-mediated ubiquitination of Rheb (Fig. 3c, d and Supplementary information, Fig. S3e). We also found that USP4 and RNF152 did not compete with each other for interacting with Rheb (Supplementary information, Fig. S3f, g). Treatment with Vialinin A, a small molecule that can inhibit the DUB activity of USP4^36^, suppressed the USP4-mediated Rheb deubiquitination and increased Rheb ubiquitination (Fig. 3e and Supplementary information, Fig. S3h). Moreover, ubiquitination of endogenous Rheb was also increased in *USP4* knockout (USP4-KO) MEFs or *USP4* knockdown cells (Fig. 3f and Supplementary information, Fig. S3i). To validate whether USP4 is a direct deubiquitinase for Rheb, we performed an in vitro deubiquitination assay using purified GST-USP4 protein. Our data confirmed that USP4 could directly deubiquitinate Rheb in vitro (Fig. 3g). These results together indicate that USP4 directly deubiquitinates Rheb.

### Growth factors regulate Rheb ubiquitination through USP4 phosphorylation

Since our data showed that Rheb ubiquitination was inhibited by EGF treatment (Fig. 1i), we examined whether the interaction between USP4 and Rheb was affected by EGF treatment and found that EGF treatment promoted their interaction at both endogenous and exogenous level (Fig. 3h and Supplementary information, Fig. S3j). It has been reported that growth factor could induce the phosphorylation of USP4 by AKT^35^. To examine whether the phosphorylation of USP4 at Ser445 affects the interaction between USP4 and Rheb, we generated a USP4 dephospho-mimetic mutant (USP4-S445A) and a phospho-mimetic mutant (USP4-S445D). Interestingly, our data showed that USP4-S445A barely bound to Rheb (Fig. 3i). In contrast, the association between Rheb and USP4-S445D mutant could be easily detected (Fig. 3i). Consistently, ectopical expression of USP4-WT and USP4-S445D, but not USP4-S445A, reduced the ubiquitination level of Rheb induced by TSC2/RNF152 (Fig. 3j and Supplementary information, Fig. S3k). Moreover, AKT inhibitor (MK2206) disrupted the interaction between USP4 and Rheb (Supplementary information, Fig. S3l), and promoted Rheb ubiquitination (Supplementary information, Fig. S3m). Taken together, these findings indicate that EGF regulates Rheb deubiquitination by inducing USP4 phosphorylation at S445.

### Ubiquitination of Rheb inhibits its activation

Ubiquitination is an essential posttranslational modification that is involved in a wide variety of cellular functions. For instance, ubiquitination not only functions as a proteolytic signal by targeting substrates for proteasome-mediated degradation, but also serves as a signal to regulate the activity of the target protein^37,38^. Since our data showed that neither RNF152 nor TSC2 could affect Rheb protein stability (Supplementary information, Fig. S3n, o). Moreover, we did not find significant differences in the half-lives of Rheb-WT and a variety of Rheb mutants such as Rheb-S16H, Rheb-S20N, Rheb-D60V, and Rheb-D60K (Supplementary information, Fig. S3p, q). These data indicate that the mono-ubiquitination of Rheb is not involved in its degradation.

Given that Rheb is a small GTPase that can convert from GDP- to GTP-bound form and thereby activates mTORC1 in response to growth factors^15,16^, we investigated whether Rheb ubiquitination has any effect on its activation using GTP pull down assay^26,39–41^. We also applied an antibody that specifically targets the active Rheb, which is widely used to detect Rheb activation^42–44^. Our data showed that knockdown of *RNF152* induced Rheb activation under the basal conditions (Supplementary information, Fig. S3r). Consistently, Rheb activity was higher in *RNF152*^*−/−*^ primary MEFs than that of control cells (Fig. 3k and Supplementary information, Fig. S3s). In addition, Rheb-K8R showed stronger binding to GTP than Rheb-WT. Moreover, TSC2 failed to inactivate Rheb-K8R, suggesting that ubiquitination negatively regulates Rheb activation (Supplementary information, Fig. S3t).

We next examined whether USP4 is involved in Rheb activation. Our data showed that depletion of USP4 as well as inhibition of USP4 activity by Vialinin A, reduced Rheb activity (Fig. 3l, m and Supplementary information, Fig. S3u). These data indicate that the deubiquitinase activity of USP4 is required for Rheb activation. Meanwhile, expression of USP4-WT and USP4-S445D, but not USP4-S445A, promoted Rheb activation (Fig. 3n). Interestingly, depletion of USP4 or Vialinin A treatment had little effect on the activation of Rheb-K8R (Supplementary information, Fig. S3v, w). In this context, we conclude that ubiquitination of Rheb by RNF152 negatively regulates Rheb activation and USP4 positively regulates Rheb activation by promoting Rheb deubiquitination in an EGF-sensitive manner (Fig. 3o).

### Ubiquitination of Rheb promotes its interaction with TSC2

**Fig. 4:**
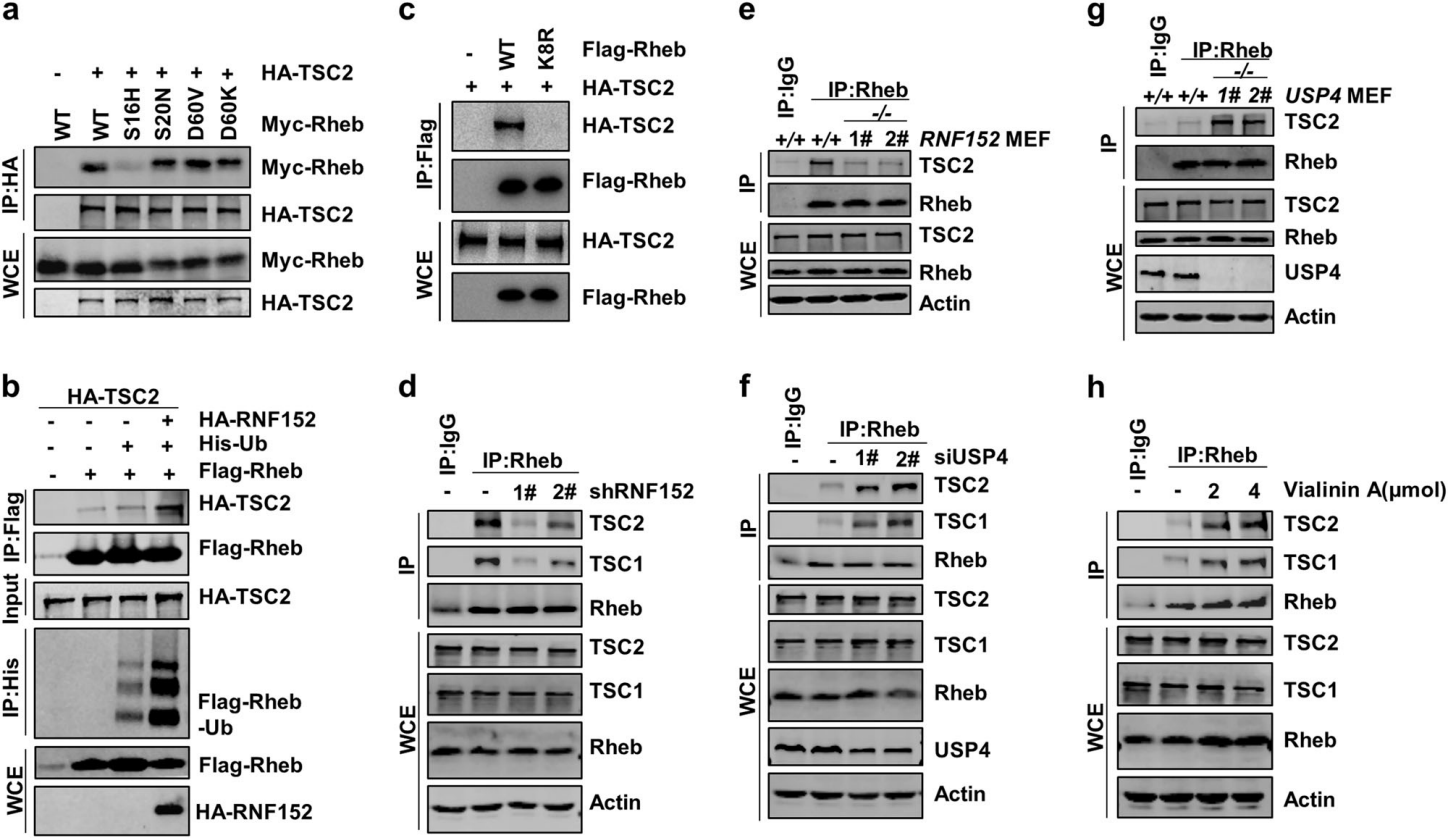
Ubiquitinated Rheb promotes its interaction with TSC. **a** Co-IP assay was performed to analyze the binding of TSC2 to different forms of Rheb mutants (active form: S16H; inactive form: D60V, D60K, S20N). **b** TSC2 purified from HEK293T cells showed stronger binding affinity to the ubiquitinated Rheb. **c** Rheb-K8R mutant showed weaker binding affinity to TSC2 as compared with Rheb-WT. **d**, **e** *RNF152* Knockdown (**d**) or knockout (**e**) reduced the binding bewteen Rheb and TSC2. The knockdown efficiency was detected by RT-PCR in Supplementary information, Fig. S2e. RNF152-KO MEF cells were analyzed via genomic DNA PCR in Supplementary information, Fig. S3s. **f**–**h** *USP4* knockdown (**f**), knockout (**g**) or inactivation of USP4 by Vialinin A (**h**) promoted the binding of Rheb to TSC2

We next examined the mechanism by which Rheb activity was inhibited by its ubiquitination. Previous study indicates that TSC complex serves as a GAP to inactivate Rheb GTPase^17^. We thus examined whether Rheb ubiquitination affects its interaction with TSCs. Consistent with  the previous study^45^, we found that TSC2 preferentially bound to the inactive form of Rheb (Rheb-S20N and Rheb-D60V) rather than the active form of Rheb (S16H) (Fig. 4a). Moreover, our data from the pull-down assay in which the immunoprecipitates extracted from HEK293T cells indicated that the ubiquitinated Rheb possessed an increased ability of binding to TSC2 (Fig. 4b and Supplementary information, Fig. S4a). The Rheb-K8R mutant exhibited much weaker binding affinity to TSC2 compared with Rheb-WT (Fig. 4c). These data together suggest that Rheb ubiquitination at K8 enhances the binding of Rheb to TSC2.

We next examined whether RNF152 affects the interaction between TSC and Rheb. Indeed, we found that the interaction between Rheb and TSC was reduced when RNF152 was depleted (Fig. 4d, e). In line with this, RNF152, but not the E3 ligase dead mutant of RNF152 (RNF152-CS), increased the binding of Rheb to TSC2 (Supplementary information, Fig. S4b). In addition, the ectopical expression of USP4, but not the deubiquitinase enzyme dead mutant USP4-CS, blocked the interaction between Rheb and TSC2 (Supplementary information, Fig. S4c). On the other hand, their endogenous interaction was increased upon depletion of USP4 or Vialinin A treatment (Fig. 4f–h). Meanwhile, deficiency of USP4 had little effect on the interaction between Rheb-K8R and TSC2 (Supplementary information, Fig. S4d).

Recent studies indicate that the TSC complex is recruited to the lysosome where the complex inhibits Rheb activation by interaction with Rheb^45^. Our data showed that the co-localization of TSC2 and LAMP2 was reduced in *RNF152* knockdown cells (Supplementary information, Fig. S4e), and the opposite results were observed in *USP4* knockdown cells (Supplementary information, Fig. S4f). Together, these data demonstrated that RNF152-mediated ubiquitination of Rheb strengthens its binding to TSC2 and thereby inhibits Rheb activation under the growth factor deficient conditions. Meanwhile, RNF152-mediated Rheb ubiquitination is dependent on TSC2-regulated inactivation of Rheb, which forms a feedback loop to regulate Rheb activity. In parallel, USP4 removes ubiquitin from Rheb and promotes the dissociation of TSC from Rheb, which ultimately activates Rheb upon the growth factor treatment (Supplementary information, Fig. S4g).

### Ubiquitination of Rheb negatively regulates mTORC1 activation

**Fig. 5:**
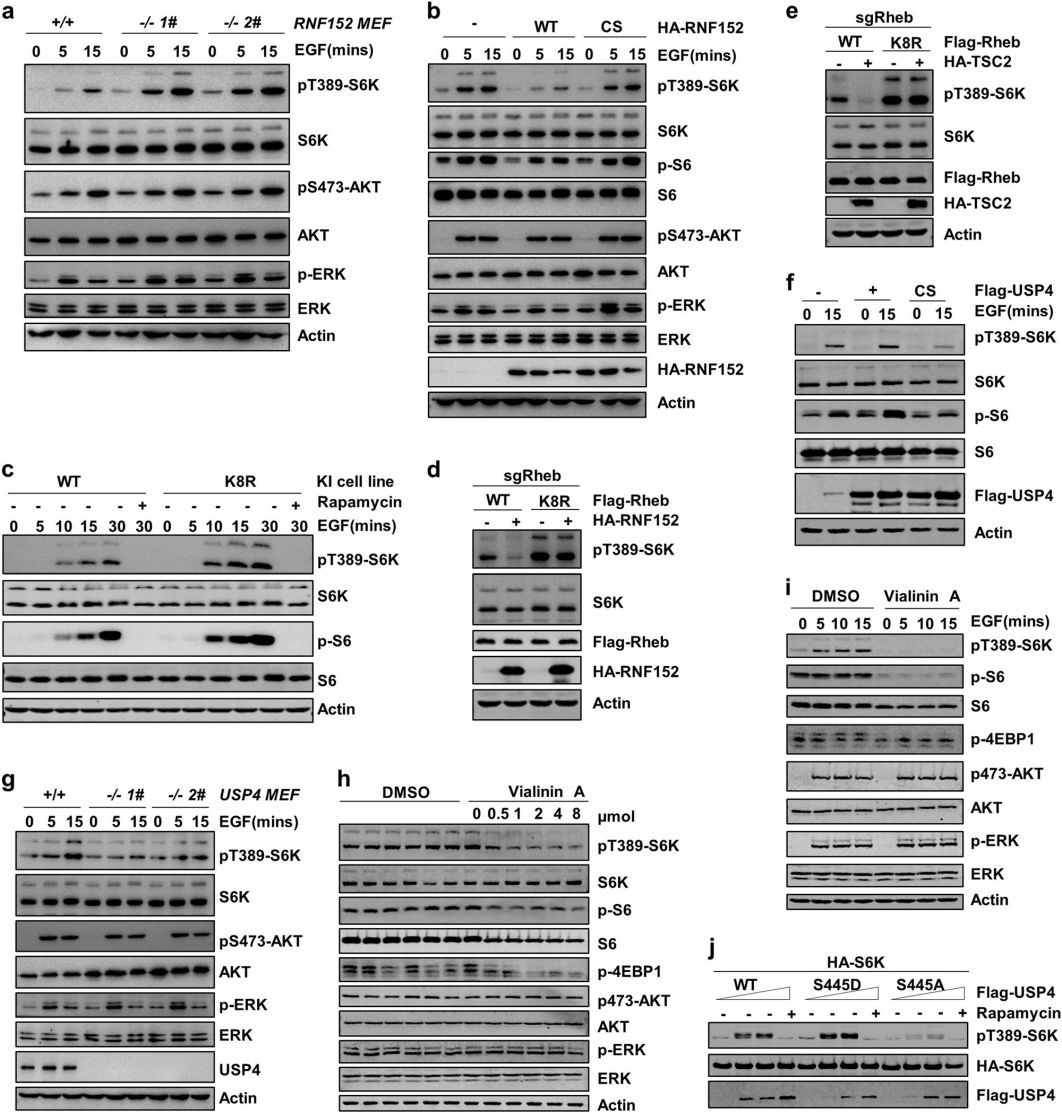
RNF152 and USP4 regulate mTORC1 activation. **a** EGF-induced mTORC1 activation was measured in primary WT or RNF152^−/−^ MEF cells. *RNF152* knockout was analyzed via genomic DNA PCR in Supplementary information, Fig. S3s. **b** Overexpressing RNF152, but not the E3 ligase dead mutant RNF152-CS, reduced EGF-induced mTORC1 activation in HEK293T cells. **c** The activation of mTORC1 induced by EGF was analyzed in Rheb-WT or Rheb-K8R knockin HEK293T cells. **d**, **e** Activation of mTORC1 induced by Rheb-K8R was resistant to RNF152 (**d**) and TSC2 (**e**). **f** Overexpressing USP4-WT, not USP4-CS, enhanced EGF-induced mTORC1 activation. **g** EGF-induced mTORC1 activation was detected in WT and USP4 deficient primary MEF cells. **h**, **i** mTORC1 activation was measured after treatment with different doses of Vialinin A for 4 h in HEK293T cells. **j** The effects of different USP4 mutants (USP4-WT, S445A, S445D) on mTPRC1 activation

Previous studies have uncovered a crucial role of Rheb in mTORC1 activation in response to growth factor^1,17^. We therefore examined whether Rheb ubiquitination regulates mTORC1 activation by detecting the phosphorylation level of S6K, S6, and/or 4EBP1. Our data showed that deficiency of RNF152 in MEFs enhanced EGF-induced mTORC1 activity (Fig. 5a). On the other hand, ectopical expression of RNF152, but not the E3 ligase dead mutant RNF152-CS, suppressed EGF-induced mTORC1 activation (Fig. 5b and Supplementary information, Fig. S5a). Consistently, Rheb-K8R displayed stronger ability to activate mTORC1 than WT-Rheb in Rheb-deficient cells upon EGF treatment (Supplementary information, Fig. S5b). In addition, Rheb-K8R knockin cells displayed stronger ability to activate mTORC1 than Rheb-WT cells upon EGF treatment (Fig. 5c). Moreover, Rheb-K8R-mediated mTORC1 activation was resistant to the inhibitory effects of both TSC2 and RNF152 (Fig. 5d, e), indicating that TSC2/RNF152-mediated Rheb ubiquitination negatively regulates mTORC1 activation in an EGF-sensitive manner.

Considering Rheb ubiquitination could be reversed by USP4, we examined whether USP4 has any effect on EGF-induced mTORC1 activation. Our data showed that overexpression of USP4-WT, but not USP4-CS, promoted the EGF-dependent mTORC1 activation (Fig. 5f). Depletion of USP4 in various cells reduced the EGF-induced mTORC1 activation (Fig. 5g and Supplementary information, Fig. S5c, d and e). Meanwhile, reintroducing USP4 WT, but not the enzymatic dead mutant (USP4-CS), in USP4-deficient cells rescued the mTORC1 activation (Supplementary information, Fig. S5f). In addition, treatment with USP4 inhibitor, Vialinin A, almost completely blocked EGF-induced the phosphorylation of S6K, S6, and 4EBP1 in a dosage- and time-dependent manner (Fig. 5h, i and Supplementary information, Fig. S5g). In parallel, USP4-S445A that lost the ability to deubiquitinate Rheb, failed to induce mTORC1 activation (Fig. 5j). Furthermore, the inhibition of mTORC1 by the depletion of USP4 was abolished by the knockdown of *TSC2* (Supplementary information, Fig. S5h), indicating that USP4 regulates EGF-induced mTORC1 activation in a TSC2-dependent manner.

### RNF152 and USP4 regulate cellular autophagy, cell proliferation, and cell size via Rheb ubiquitination

**Fig. 6:**
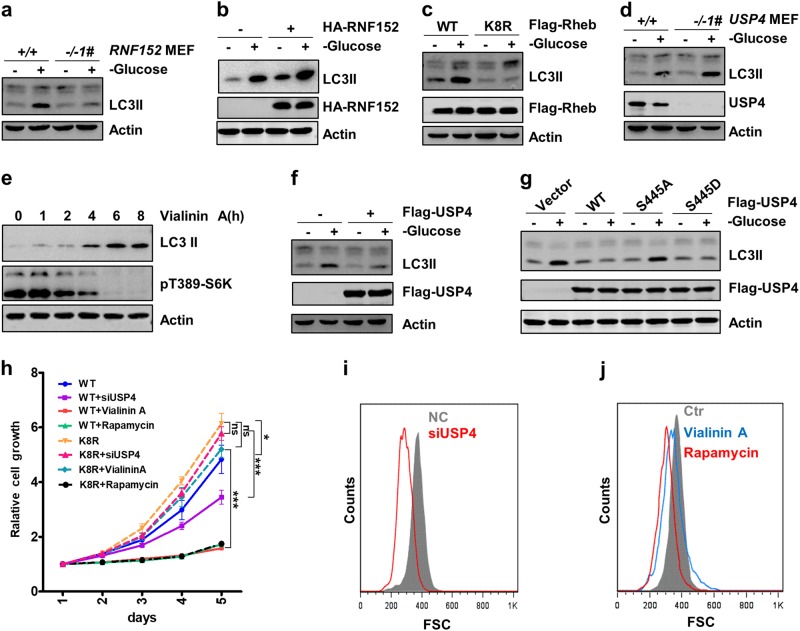
Rheb ubiquitination regulates cell autophagy, cell proliferation, and cell size. **a** Cell autophagy induced by glucose starvation was analyzed in WT and *RNF152*^−/−^ MEF cells. Cells were harvested after treated with Bafilomycin A1 (100 nM) for 4 h. RNF152-KO MEF cells were analyzed by genomic DNA PCR in Supplementary information, Fig. S3s. **b** The protein levels of LC3II were analyzed in HEK293T cells after glucose starvation and Bafilomycin A1 treatment as in (**a**). **c** Under glucose starvation, Rheb-K8R showed stronger blocking effects on autophagy than Rheb-WT. Bafilomycin A1 treatment was performed as in (**a**). **d** *USP4*^−/−^ MEF cells showed higher autophagy levels than *USP4*^*+/+*^ MEF cells. Cells were treated with Bafilomycin A1 treatment as (**a**). **e** The effects of USP4 on cell autophagy were analyzed by examining the LC3II protein levels. Cells were treated with Vialinin A (2 μmol) for indicated time periods together with Bafilomycin A1 as in (**a**). **f**, **g** The effects of WT or USP4 mutants on autophagy were detected in HEK293T cells. **h** *USP4* knockdown and Vialinin A treatment blocked cell proliferation in Rheb-WT-expressed, but not Rheb-K8R expressed SW620 cells. Data were analyzed by two-way ANOVA. *P* < 0.05 was considered statistically significant. *P < 0.05 and ***P < 0.001, respectively, ns not significant. **i**, **j** Cell size was measured in *USP4* knockdown cells (**i**) and Vialinin A-treated cells (**j**)

Next, we determined whether Rheb ubiquitination affects the cellular functions of mTOR signaling in response to various environmental stresses. Because mTORC1 is a negative regulator of autophagy^46^, we examined whether the RNF152-suppressed- and USP4-potentiated- mTORC1 activation affects cellular autophagy. Our data showed that depletion of RNF152 resulted in a reduced level of LC3II (Supplementary information, Fig. S6a). Moreover, autophagy was inhibited in RNF152-depleted MEFs and could be promoted by RNF152 overexpression in HEK293T cells evidenced by an increase of LC3II (Fig. 6a, b and Supplementary information, Fig. S6a). Consistently, the expression of Rheb-K8R resulted in a stronger suppression of autophagy as compared with the expression of Rheb-WT (Fig. 6c). Taken together, these data suggest that RNF152-mediated Rheb ubiquitination regulates cellular autophagy.

We also examined whether USP4 is involved in the regulation of mTORC1-mediated cellular function. We found that knockdown or knockout of *USP4* led to a sharp increase of LC3II level (Fig. 6d and Supplementary information, Fig. S6b). Accordingly, treatment with Vialinin A decreased the levels of p62 and the phosphorylated ULK1 (Fig. 6e and Supplementary information, Fig. S6c). Moreover, expression of USP4-WT, but not USP4-S445A, suppressed cellular autophagy (Fig. 6f, g).

Since mTORC1 is a master regulator of cell proliferation and cell size^1,47,48^, we examined the biological significance of USP4 in the regulation of cell proliferation and cell size. We found that depletion of USP4 or treatment of Vialinin A suppressed cell proliferation. On the other hand, expression of Rheb-K8R promoted cell proliferation and abolished the inhibitory effect on cell proliferation induced by siUSP4 and Vialinin A (Fig. 6h). Consistently, knockdown of *USP4* or Vialinin A treatment reduced the cell size (Fig. 6i, j). These data suggest that USP4 is a positive regulator of mTORC1-mediated cellular activities including the control of cellular autophagy, cell proliferation, and cell size.

### RNF152 and USP4 regulate tumor growth in an mTORC1-dependent manner in vivo

**Fig. 7:**
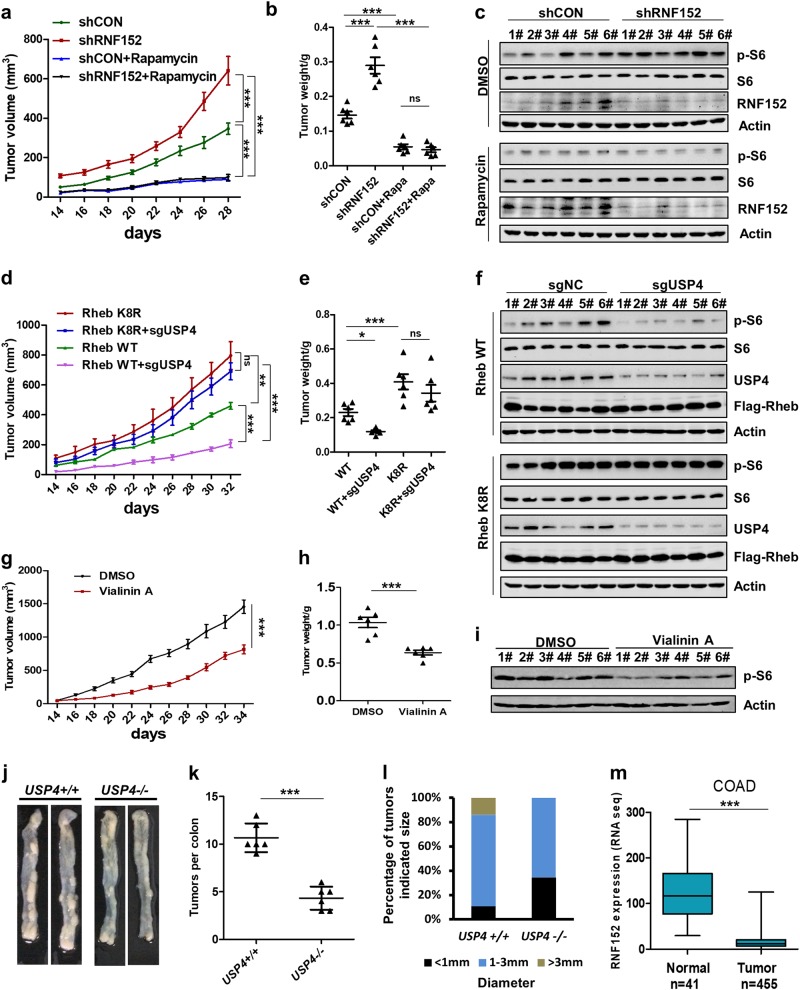
Regulation of tumor growth by RNF152 or USP4 in an mTOR-dependent manner. **a**, **b** *RNF152* knockdown promoted SW620 tumor cell growth in a xenograft model (*n* = 6 per group). The diameter of the tumor was measured every 2 days after 14 days of injection. Tumors were obtained on the 28th day after injection and the weight of the tumors were measured (**b**). Data were analyzed by two-way ANOVA (**a**) or one-way ANOVA (**b**). *P* *<* 0.05 was considered statistically significant. ***P < 0.001. ns not significant. **c** mTORC1 activation was tested in the tumor samples. **d**–**f** Rheb-WT or Rheb-K8R mutant was expressed stably in control or USP4 deficient SW620 cells and then the cells were injected into nude mice subcutaneous. The volume (**d**) and weight (**e**) of tumors, and the mTORC1 activation (**f**) in tumor samples were shown. Data were analyzed by two-way ANOVA (**d**) or one-way ANOVA (e) *P* *<* 0.05 was considered statistically significant. *P < 0.05, **P < 0.01, and ***P < 0.001, respectively. ns not significant. **g**, **h** The effects of Vialinin A on tumor growth were examined by injecting HCT116 cells into nude mice (*n* = 6 per group). Data were analyzed by two-way ANOVA (**g**) or Student’s *t* test (**h**). *P* *<* 0.05 was considered statistically significant. ***P < 0.001. **i** The effect of Vialinin A on the phosphorylation of S6 was examined in tumor samples from the xenografts of nude mice induced by the injection of HCT116 cells. **j** Representative images of colon tumors in mice on the 60th day after injection of azoxymethane. **k**, **l** Number (**k**) and size (**l**) of colon tumors in wildtype (*n* = 6) and *USP4*^*−/−*^ (*n* = 6) mice. Data were analyzed by Student’s *t-*test (**k**). *P* *<* 0.05 was considered statistically significant. ***P < 0.001. **m** The expression level of RNF152 in colon cancers based on the TCGA database. Data were analyzed by Student’s *t-*test. *P* *<* 0.05 was considered statistically significant. ***P < 0.001,

We next examined whether RNF152 and USP4 are involved in mTORC1-mediated tumorigenesis in vivo. Our data showed that SW620 cells lacking RNF152 exhibited strong ability to form tumor with a rapid growth rate, which could be abolished by rapamycin treatment (Fig. 7a, b and Supplementary information, Fig. S7a). Western blotting for p-S6 demonstrated that knockdown of *RNF152* resulted in the mTORC1 activation in tumors (Fig. 7c). Meanwhile, knockdown of *USP4* led to reduced tumor size, as well as reduced expression level of p-S6, which could be rescued by the expression of Rheb-K8R (Fig. 7d–f; Supplementary information, Fig. S7b). In addition, USP4 inhibitor, Vialinin A, inhibited tumor growth and reduced the phosphorylation level of p-S6 (Fig. 7g–i; Supplementary information, Fig. S7c).

To confirm that USP4 is involved in tumorigenesis, we generated *USP4* knockout mice and investigated the role of USP4 in colorectal cancer using the established mouse model of colitis-associated colorectal tumorigenesis. We intraperitoneally injected wildtype and *USP4*^−/−^ mice with azoxymethane followed by three rounds of DSS treatment. By counting the number of tumors in mouse colons, we found that *US*P4^−/−^ mice developed fewer tumor colonies compared with *WT* mice (Fig. 7j, k and l), whereas the body weight loss of *WT* mice was higher than that of *USP4*^−/−^ mice (Supplementary information, Fig. S7d). Consistently, mTORC1 activity was decreased in the tumor samples from *USP4*^−/−^ mice (Supplementary information, Fig. S7e).

In an attempt to clarify the biological significance of RNF152 in tumor growth, we analyzed the expression levels of RNF152 by searching TCGA database and found that RNF152 is down-regulated in various types of cancers, including colon, lung, kidney, and liver cancers (Fig. 7m and Supplementary information, Fig. S7f, g and h). In summary, these data indicate that both RNF152 and USP4 play important roles in tumor growth via regulating mTORC1 activation (Supplementary information, Fig. S7i).

## Discussion

Great attention has been drawn in studies of the regulatory network of mTORC1 activation since its pivotal roles in severe diseases have been confirmed by the accumulating evidence. Recent studies indicate that mTORC1 is tightly controlled by ubiquitination. However, whether any type of ubiquitination regulates Rheb-mediated mTORC1 activation is unclear. In this study, we found that mono-ubiquitination is an important regulatory mechanism for Rheb activity.

Small GTPases are usually activated by GEFs, while their GTP hydrolysis is facilitated by GAPs. Such a “GTPase cycle” is ongoing at molecular and cellular levels^33,49,50^. Accumulating evidence reveals that mono-ubiquitination mediates cellular activities by recruiting different effectors^20^. For instance, E3 ligase Rad18 interacts with Rad6 to regulate PCNA mono-ubiquitination responding to DNA damage repair via recruitment of DNA polymerases^21^. Besides, the mono-ubiquitination of H2AX driven by TRAF6 is a prerequisite for recruiting ATM^22^. In the current study, we offered the evidence showing that such “GTPase cycle” can be disrupted by Rheb mono-ubiquitination. Several lines of evidence from our data revealed that Rheb ubiquitination is dependent on TSC2 (Fig. 1a–f). Thus, we conclude that the TSC complex not only functions as a GAP for Rheb, but also regulates Rheb ubiquitination via its conjunction with the E3 ligase RNF152 (Figs. 2h, 2k). However, we cannot exclude the possibility that TSC has its own E3 ligase activity, which deserves an overall and systematic investigation in the future.

We noticed that the ubiquitination patterns of Rheb are different under in vivo and in vitro conditions. One possibility is that the RNF152-mediated Rheb ubiquitination may also be regulated by some unrecognized mechanisms, such as other unknown binding partners, unidentified protein modifications, or their subcellular localization. It is also possible that the in vitro ubiquitination assay we used is not as same as in vivo assay. For example, it is still unclear whether the broad-spectrum ubiquitin-conjugating enzyme E2 (UBC5a) we used in our in vitro assay is the same as the E2 that promotes Rheb ubiquitination in vivo. We also noticed that the endogenous ubiquitination of Rheb mainly appears at a single band (Figs. 1c, 1h), whereas exogenous experiment showed multiple bands for ubiquitinated Rheb. We think the reason is that the endogenous ubiquitination of Rheb is weaker than Rheb ubiquitination in cells ectopically expressing Rheb. Moreover, our data indicate that the mono-ubiquitination of Rheb might occur on multiple sites while Rheb K8 is the major mono-ubiquitination site targeted by RNF152. However, whether Rheb ubiquitination occurs at other sites needs to be addressed in future studies.

Our previous study showed that RNF152 can mediate K63-linked polyubiquitination of RagA, which recruits GATOR1 and consequently inactivates the mTORC1 signaling in response to amino-acid deprivation^25^. In the current study, we identified Rheb as another substrate of RNF152 and found that RNF152 acts as a negative regulator of Rheb activation by targeting Rheb for ubiquitination in response to growth factor starvation. Together with these findings, we conclude that RNF152 is a negative regulator of both amino acid- and growth factor-induced mTORC1 activation.

Furthermore, we demonstrated that Rheb ubiquitination strengthens the interaction between Rheb and the TSC complex (TSC1/2) (Fig. 4). Co-IP assay demonstrated that the ubiquitinated Rheb possesses an increased ability of binding to TSC2 (Fig. 4b and Supplementary information, Fig. S7a). This is consistent with the fact that TSC2 preferentially bound to the inactive form of Rheb (Rheb D60V), which can be highly ubiquitinated (Figs. 4a, 1k). Thus, we conclude that Rheb ubiquitination provides a regulatory platform for the interaction between TSC2 and Rheb. However, the detailed mechanism by which Rheb ubiquitination affects its binding to TSC2 remains unknown. Although we did not find any typical ubiquitin-interacting motif (UIM) in the TSC complex, it is possible that UIM exists either inside the three-dimensional structure of TSC1, TSC2, or TBC1D7, or in other unidentified binding partners of the TSC complex. Moreover, we cannot rule out the possibility that some unidentified ubiquitin-binding protein might be recruited by the TSC complex, thereby enhancing the binding affinity of TSC2 to the ubiquitinated Rheb.

Protein ubiquitination is a reversible process^34,37,51^. We found that USP4 is an enzyme that specifically removes ubiquitin moiety from Rheb. Interestingly, we also found that overexpression of other DUBs such as USP5, USP11, USP15, or USP22, could target Rheb for deubiquitination (Supplementary information, Fig. S3a). Our unpublished data showed that deubiquitination of Rheb by USP5, USP11, USP15, or USP22 is independent of USP4, suggesting Rheb ubiquitination is regulated by multiple DUBs other than USP4. Moreover, we found that USP4 is involved in the activation of both Rheb and mTORC1 in response to EGF treatment (Figs. 3l, 5g). Meanwhile, we found that *US*P4^*−/−*^ mice developed fewer tumors as compared with *US*P4^*+/+*^ mice using a colitis-associated colorectal tumorigenesis mouse model (Figs. 7j, 7k and 7l). Vialinin A, an inhibitor of USP4, inhibited the tumorigenesis (Figs. 7g, 7h). These data indicate that USP4 plays an important role in tumor growth via mTORC1 activation, which may shed light on the development of new diagnostic markers and therapeutic strategies for cancer treatments.

In summary, our results revealed an effective regulatory system and a rational in vivo mechanistic model of mTORC1-activated tumorigenesis determined by the exquisitely regulated processes of ubiquitination and deubiquitination of Rheb, which may facilitate future study of cancer treatments.

## Materials and Methods

### Antibodies and reagents

The anti-Flag, anti-HA, anti-LC3II (L7543), anti-p62 (L0067) primary antibodies and secondary antibodies were obtained from Sigma. Anti-ubiquitin (ab19247), anti-GST (ab19256) antibodies were purchased from Abcam. Anti-c-Myc (9E10 sc-40) antibody was obtained from Santa Cruz Biotechnology (Santa Cruz, CA). The antibodies against p-S6K (9234 s/L), p-S6 (4858 s), p-ULK (6888 s), mTOR (2983 s), S6K (9202 s), S6 (2217 S), p-4EBP1 (2855 S), Hamartin/TSC (16935), TSC2 (4308 S), ERK1/2 (4695), phospho-ERK1/2 (4370), Ki-67 (12202 S), and TβR1 (3712 S) were obtained from CST. Antibody against Rheb (H00006009-M01) was obtained from Abnova. Anti-USP4 (A300–830A) was purchased from BETHYL. Glutathione SepharoseTM 4B (17–0756–01) was obtained from GE Healthcare. NI-NTA Agarose (30210) was purchased from QIAGEN. DMEM, FBS, amino acids (50×), β-mercaptoethanol, penicillin, and streptomycin were purchased from Gibco. DMEM (amino acid-free) was purchased from Genetimes Technology, Immobilized γ-Amino-hexyl-GTP (AC-117L) was obtained from Jena Bioscience. RheB Activation Assay Kit was purchased from NewEast Biosciences. Rapamycin (V900930), Azoxymethane (A5486), and Rheb (SAB4200517) were purchased from Sigma. Vialinin A (858134–23–3) was obtained from TOCRIS Bioscience. DSS (160110) was purchased from MP Biomedicals, LLC. MTT (3-[4,5-dimethylthiozol-2-yl]-2,5-diphenyl tetrazolium bromide T0793) was purchased from Sangon Biotech, Shanghai, China.

### Cell culture and derivation of MEFs

HEK293T, HCT116, and MEF cells were cultured in DMEM supplemented with 10% fetal bovine serum at 37 °C in 5% CO2. H1299 and SW620 cells were cultured in RPMI 1640 supplemented with 10% fetal bovine serum at 37 °C in 5% CO2. RNF152 or USP4 KO MEFs cells were generated as described previously. Briefly, mice homozygous for RNF152 or USP4 were intercrossed. The pregnant female mice were killed at day 13 post-coitum. The individual embryos were collected, and any extra-embryonic tissue was removed. Then, the embryos were dispersed using scissors, and the dispersed tissues were trypsinized at 37 °C for 30 min. Trypsin was inactivated by adding DMEM. The cells were isolated via centrifugation at 4000 rpm in a microcentrifuge for 5 min at room temperature. Then, the cells were resuspended in DMEM and were seeded on 10 cm dishes.

### Generation of the USP4 KO HEK293T cell lines

*USP4* knockout cell lines were generated using lentiCRISPR methods^52^. Briefly, guide RNA (sgRNA) was constructed into the lentiviral expression vector with Cas9 and sgRNA (lentiCRISPR). The lentiCRISPR vector was linearized using BsmBI. The sequences of sgRNAs are:

sgRNA USP4-1#: CTGCCGTGAGCGACCGGATG.

sgRNA USP4-2#: GAGGACCACACTCCAACGCG.

### Generation of Rheb K8R knockin HEK293T cell lines

Rheb-K8R knockin HEK293T cells were generated using CRISPR/Cas9. Rheb-K8R-specific sgRNA oligos were designed by the CRIPSR website (http://crispr.mit.edu/), the targeting sequence at K8 locus is 5′- GATCGCGATCTTCCGGGACT-3′, the oligo donor sequence is 5′- ggccggggctgaggaggccgccaagatgccgcagtccaagtcccggcgaattgccatactgggctaccggtctgtgggtgagtggccggtggccgcgcggcctcctcgcgccgccggggcctcgcctgggagccg -3′. To generate Rheb-k8R knockin cell lines, the donor was inserted into pCDNA3.1 vector. 2 μg sgRNA and 2 μg 135-bp homology arm-containing donor plasmid were transfected into HEK293T cells. 24 h later, cells were treated with puromycin (2 μg/ml). Then, the remained cells were separated into 96-well plate. After genomic DNA collection, PCR was performed and the products were sequenced.

### Plasmids and virus

The His-ubiquitin expression plasmids were constructed as previously reported^53^. The HA-S6K, Rheb-WT, and Rheb mutant plasmids (Rheb^S16H^, Rheb^S20N^, Rheb^D60K^, Rheb^Q64L^) were kindly provided by Dr. Kunliang Guan. RNF152 or USP4 and its mutants were cloned into pCDNA3.1 vector and a Flag, or an HA tag was fused to the N terminus. GST-tagged RNF152 and its mutants were cloned into pGEX-4T-2 vector. shRNAs were cloned into the Migr-Venus-Mir30 vector. All of the constructs were confirmed via DNA sequencing.

### Virus preparation and viral infection

To prepare retrovirus for the knockdown experiments, HEK293T cells were transfected with the Migr-Venus-shRNA vector and the retrovirus packaging vectors Gag and pCMV-VSVG using the calcium phosphate-DNA co-precipitation method. To prepare lentivirus for overexpression, the HEK293T cells were transfected with PLVX-Flag-IRES-ZsGreen and the packaging vectors PSPAX2 and pMD2G using the calcium phosphate-DNA co-precipitation method. Viral infection was performed as previously described^53^. Briefly, medium containing the virus was collected 48 h after transfection. HEK293T, H1299, MEFs, SW620, and HCT116 cells were cultured in the collected viral supernatant in the presence of polybrene.

### siRNA knockdown

Non-specific control siRNA and siRNAs for USP4 and TSC1/2 were purchased from GenePharma. Cells were transfected with siRNA oligonucleotides using Lipofectamine. siRNA transfection of cells was performed according to the manufacturer’s instructions. The following siRNAs were used:

siUSP4-1#: 5′- AACATGTCCGAGTTTGTCTGT -3′,

siUSP4-2#: 5′- AACTGTAAGAAGCATCAACAG -3′,

siUSP4-3#: 5′- TTAAACAGGTGGUGAGAAA -3′,

siUSP4-4#: 5′- CGAAGAATGGAGAGGAACA -3′

siTSC1: 5′-CCGGACAGTGTTGGACAGCTA-3′,

siTSC2-1#: 5′-AAGGATTACCCTTCCAACGAA-3′,

siTSC2-2#: 5′-CGACGAGTCAAACAAGCCAAT-3′.

sgTSC2-1#: 5′-CACCGGGTGGCCAGCTTTCGGACC-3′,

sgTSC2-2#: 5′-CACCGAGACCACCAGGTCCGAAAGC-3′,

To generate shRNA vectors, double-stranded DNAs for shRNF152 (5′-GCUCUGUAGACAAGUAGAATT-3′ and 5′-GCUGGAAUGUCAGAUCUGUTT-3′) were cloned into the Migr-Venus-Mir30 vector.

### RT-PCR

Total mRNA was isolated using TRIzol (Invitrogen), and 1 μg of RNA was used to synthesize cDNA using the Prime Script^TM^ RT reagent kit (Takara, DRR037A) according to the manufacturer’s protocol. PCR was performed using a standard protocol.

### Growth factor, amino acid, and glucose starvation and re-stimulation

For EGF-stimulation, cells were treated as previously described^10^. In brief, cells were rinsed with PBS and incubated in serum-free DMEM medium for 24 h. Then, cells were stimulated with EGF for indicated time periods. For amino acid starvation, cells were incubated in amino acid-free DMEM for 50 min and then stimulated with amino acids for indicated time periods. For glucose starvation, cells were incubated in glucose-free DMEM medium for indicated time periods.

### Immunoprecipitation (IP) and western blotting

IP and western blotting were performed as previously described^53^. Transfected HEK293T cells were lysed in CHAPS lysis buffer (40 mM HEPES, pH 7.4, 120 mM NaCl, 1 mM EDTA, 10 mM β-glycerophosphate, 0.3% CHAPS, and a cocktail of proteinase inhibitors)^9^. After sonication for 10 min, the soluble fraction of the cell lysates was isolated via centrifugation at 12,000 rpm in a microcentrifuge for 15 min at 4 °C. For IP, the cell lysates were centrifuged to remove the cell debris and then were incubated with HA-conjugated beads (Abmart) or M2 beads (Sigma) for 2-3 h. Endogenous RNF152 was immunoprecipitated using an anti-RNF152 polyclonal antibody. The beads were boiled after extensive washing; resolved via SDS-PAGE gel electrophoreses, and analyzed via immunoblotting. The proteins were detected using the Odyssey system (LI-COR Biosciences).

### In vivo ubiquitination assay

For the in vivo ubiquitination assay using Ni-NTA beads^54^, the cells were transfected with His-ubiquitin. Then, the transfected cells were lysed using denaturing Buffer A (6 M guanidine-HCl, 0.1 M Na_2_HPO_4_/NaH_2_PO_4_, 10 mM imidazole, pH 8.0), and the ubiquitinated proteins were purified using Ni-NTA beads. The beads were then washed sequentially with Buffer A, Buffer B (8 M urea, 0.1 M Na_2_HPO_4_/NaH_2_PO_4_, pH 8.0, 0.01 M Tris-HCl, pH 8.0, 10 mM β-mercaptoethanol), Buffer C + 100 (Buffer C containing 0.2% Triton X-100), and Buffer C (8 M urea, 0.1 M Na_2_HPO_4_/NaH_2_PO_4_, pH 6.3, 0.01 M Tris-HCl, pH 6.3, 10 mM β-mercaptoethanol). The washed beads were incubated in 40 μl elution buffer (200 mM imidazole, 0.15 M Tris-HCl, pH 6.7, 30% glycerol, 5% SDS, 0.72 M β-mercaptoethanol) at room temperature for 30 min. The input fractions and eluates were analyzed via Western blotting.

To detect ubiquitination of endogenous Rheb under denaturing conditions, HEK293T cells were lysed in RIPA buffer (1× PBS, 1% NP-40, 0.5% sodium deoxycholate, 1% SDS, 10 mM *N*-ethylmaleimide, and a cocktail of proteinase inhibitors). Cell lysates were boiled for 10 min, diluted in 10 volumes of lysis buffer without SDS, and subjected to immunoprecipitation using anti-Ubiquitin antibody at 4 °C overnight. Endogenous ubiquitination of Rheb was detected by immunoblotting using anti-Rheb antibody.

For sequential affinity to obtain ubiquitinated Rheb, the transfected HEK293T cells were lysed using Buffer A and incubated with Ni-NTA beads, after sequentially washing, the eluted proteins were then subjected to immunoprecipitation using an anti-Myc antibody. Ubiquitination of Rheb was detected by immunoblotting using an anti-Rheb antibody.

### In vitro ubiquitination assay

To detect the in vitro ubiquitination of Rheb, His-Rheb, and GST-RNF152 were purified from *E. coli*. The in vitro ubiquitination reaction was performed using E1 (Bostone Biochem), E2, ubiquitin, GST-RNF152, His-Rheb, and 2 mM ATP in a final volume of 20 μl at 37 °C for 2 h. The ubiquitinated Rheb proteins were detected via Western blotting using an anti-Rheb antibody^55^.

### Lysosomes isolation

Lysosomes were isolated with lysosome Isolation Kit (Catalog Number LYSISO1, sigma), all subsequent steps of the lysosomal isolation were performed according to manufacturer’s description. In brief, the transfected HEK 293 T cells were harvested on ~90% confluency, and the cells were centrifuged for 5 min at 600×*g*. We then added extraction buffer to break the cells in a 7 ml Dounce homogenizer using Pestle B (small clearance). After 20 strokes, the nuclei were removed by centrifugation at 1000*×g* for 10 min, the supernatant was centrifuged at 20,000×*g* for 20 min and the resulting pellet, containing the crude lysosomal fraction (CLF). To further enrich the lysosomes in the CLF, option C was used according to the manufacturer’s protocol. The final pellet (lysosomal) fraction was prepared for immunoblotting.

### Active Rheb immunoprecipitated assay

Active Rheb was Immunoprecipitated by Rheb activation assay kit from NewEast Biosciences, all subsequent steps of the active Rheb Immunoprecipitated were performed according to manufacturer’s description. In brief, transfected HEK293T were harvested on ~90% confluency, cells were lysed in lysis buffer at 4 °C for 30 min., the soluble fraction of the cell lysates was isolated via centrifugation at 12,000 rpm in a microcentrifuge for 15 min at 4 °C. For IP the active Rheb, the cell lysate was centrifuged to remove the cell debris and incubated with protein A/G beads and anti-active Rheb monoclonal antibody for 2 h. Then the beads were washed 3 times with lysis buffer, and suspended in 40 μl SDS-PAGE sample buffer. The proteins were boiled, resolved via SDS-PAGE gel electrophoreses, and analyzed via immunoblotting. The proteins were detected using the Odyssey system (LI-COR Biosciences).

### GTP-binding assay

For binding of Rheb to GTP-Agarose beads, the transfected HEK293T cells were harvested on ~90% confluency. Cells were suspended in binding buffer (20 mM HEPES pH 8, 150 nM NaCl, 10 mM MgCl2, and a cocktail of proteinase inhibitors) and lysed using three freeze thaw cycles, then centrifuged at 14,000× *g* and the supernatants were incubated with 100 μl of GTP-Agarose suspension (G9768, Sigma Aldrich) for 1 h with rotation at 4 °C . The beads were pelleted by centrifugation, washed three times in binding buffer and suspended in 40 μl SDS-PAGE sample buffer. The proteins were boiled, resolved via SDS-PAGE gel electrophoreses, and analyzed via immunoblotting. The proteins were detected using the Odyssey system (LI-COR Biosciences).

### Immunofluorescence staining

The cells were washed three times with PBS and were fixed for 15 min at room temperature with 4% (vol/vol) paraformaldehyde and permeabilized with 0.2% Triton X-100 for 20 min on ice. Following permeabilization, nonspecific binding in the cells was blocked by incubation for 30 min at room temperature with 1% BSA in PBS and then cells were incubated overnight with specific primary antibodies. After washing by PBS for three times, cells were incubated for another 1 h with secondary antibodies (Alexa Fluor 488–conjugated anti–mouse IgG (A21202), Alexa Fluor 488–conjugated anti–rabbit IgG (A21206) or Alexa Fluor 555–conjugated anti–mouse IgG (A31570). Nuclei were counterstained with DAPI. All images were collected with a confocal microscope.

### GST pull-down assay

His-Rheb protein was purified from *E. coli* and incubated with 10 μg purified GST or GST-RNF152 protein. The GST proteins were purified using glutathione sepharose 4B, and the bound Rheb was detected via Western blotting.

### MTT cell proliferation assay

Rheb WT and Rheb K8R SW620 cells were seeded in 96-well plates at an initial cell density of 1500 cells per well in quintuplicate. Cell viability was assessed by MTT assay as previously described^53^. Briefly, 10 μl MTT (5 mg/ml) was added to each well, and plates were incubated at 37 °C for 4 h. After incubation, 200 μl dimethyl sulfoxide was added, and the color formation was quantified at 490 nm wavelength. For all statistical tests, in all the results, *P *<* 0.05, **P *<* 0.01, and ***P *<* 0.001, ns denotes not statistically significant. Each sample was performed in triplicate and each experiment was repeated at least three times independently.

### Cell size analysis

To determine cell size, H1299 cells were transfected with individual siRNAs, and the cells were seeded on 10 cm dishes until 30% confluence, followed by culturing under the indicated conditions for 72 h. The culture medium was replaced every 24 h. The cells were harvested and subjected to FACS analysis to determine cell size. The X-axis indicates the relative cell size.

### Generation of *RNF152* and *USP4* knockout mice

RNF152 KO mice were generated using CRISPR-Cas9 methods^56^. Briefly, guide RNA (gRNA) expression vectors were constructed for pGS3-T7-gRNA. The pGS3-T7-gRNA vector and the Cas9-encoding plasmids were linearized using DraI and NotI, respectively. The linearized templates were transcribed in vitro via run-off reactions using T7 RNA polymerase, the in vitro Transcription T7 Kit (Takara) and the Sp6 mMESSAGE mMACHINE Kit (Ambion), respectively. TE solution containing 25 ng/μL gRNA and 50 ng/μl Cas9 mRNA was injected into the cytoplasm of one-cell stage embryos. A mismatch-sensitive T7E1 assay was used to identify the founders. To confirm the modification in the founders, the PCR products from each founder were generated using the TA cloning kit (Takara) according to the manufacturer’s instructions. *USP4* knockout mice were generated using TALEN methods as described previously^57^. For USP4  KO mice, TALEN right arm is: 5′-ATCTTATTGACAGCCGGT-3′; left arm is: 5′-GTCAAAGCCCACATACT-3′; To confirm the frame-shift in the founders, the PCR products from each founder were generated using the TA cloning kit (Takara) according to the manufacturer’s instructions. PCR was used to identify the genotype of the offspring from the intercrossed mice.

### TCGA data analysis

Level 3 data for mRNA expression from TCGA were downloaded and processed using standard methods. mRNA expression was measured using the Illumina HiSeq 2000 RNA Sequencing version 2 program. Gene expression in normal and cancer samples was analyzed, and the data were represented as box-and-whisker plots. Statistical significance was assessed by the Wilcoxon test.

### Tumor xenografts

Six-week-old male nude mice were obtained from Shanghai Experimental Animal Center (Shanghai, China). SW620 or HCT116 cells were infected with retrovirus expressing vector, shRNF152 and shUSP4, or lentivirus-expressed Rheb-WT and Rheb-K8R, and selected with 5 μg/mL puromycin in culture medium for 2 weeks. Then SW620/HCT116 cells were trypsinized into single cell suspensions and resuspended in PBS. Approximately 5×10^6^ SW620/HCT116 cells in 100 μl were injected into the right side and left dorsal flanks of each nude mouse, respectively. From 14 days after injection, the diameter of the tumor was measured every 2 days by a vernier caliper. Rapamycin was reconstituted in absolute ethanol at 10 mg/ml and diluted in 5% Tween 80 and 5% Peg-400 before injection. Treatment was conducted by intraperitoneal injection of 1.5 mg/kg/d rapamycin for 5 consecutive days on day 6 after tumor cell injection; injections of carrier solution were used as controls. Vialinin A treatment was conducted by intraperitoneal injection of 5 mg/kg/d every other day, starting at day 6 after tumor cell injection. Tumor volume was calculated using the formula: width^2^×length×0.5. Tumor diameter must not exceed 20 mm in an adult mouse, according to the IACUC. None of the experiments exceeded this limit in our study. All results were presented as mean ± SEM and analyzed by two-tailed *t*-test. *P *<* 0.05; **P *<* 0.01; ***P *<* 0.001; ns not statistically significant.

### Azoxymethane–DSS model of colorectal tumorigenesis

Male (USP4^+/+^, *n* = 6; *USP4*^−*/−*^, *n* = 6) mice used were at the age of 6 weeks. For cohousing experiments, wild-type and knockout mice were co-housed for 2 weeks before injection of azoxymethane (AOM). Mice were injected intraperitoneally with 10 mg of AOM (sigma) per kg body weight, as described previously^58^. After 5 days, 2% DSS was given in the drinking water for 6 days followed by regular drinking water for 2 weeks. This cycle was repeated twice with 1.5% DSS. Mice were killed on day 60, and the tumors were counted and photographed. Total number of tumors per colon in mice were analyzed.

### Quantification and statistical analysis

GraphPad Prism 5 software was used for data analysis. All experiments were repeated at least three times. Data were shown as mean ± SEM. Pairwise statistical significance was evaluated by two-tailed Mann–Whitney *U*-test or Student’s *t*-test. Statistical significance between multiple groups was evaluated by one-way ANOVA or two-way ANOVA with Fisher’s LSD test or Bonferroni test. *P* value was considered statistically significant. In the graphed data *P values  < 0.05, **P values  < 0.01, and ***P values  < 0.001, respectively. ns not significant.

## Electronic Information

Supplementary information, Fig. S1

Supplementary information, Fig. S2

Supplementary information, Fig. S3

Supplementary information, Fig. S4

Supplementary information, Fig. S5

Supplementary information, Fig. S6

Supplementary information, Fig. S7
