# Supplementary material for: Ubiquitination of Rheb governs growth factor-induced mTORC1 activation
Source: Cell Res. 2018 Dec 4;29(2):136–50. doi: 10.1038/s41422-018-0120-9 (PMC6355928; doi:10.1038/s41422-018-0120-9)
Supplement: Supplementary file 1 — Supplementary information, Fig. S1 [file 41422_2018_120_MOESM1_ESM.docx]

**Supplementary information, Fig. S1**

**
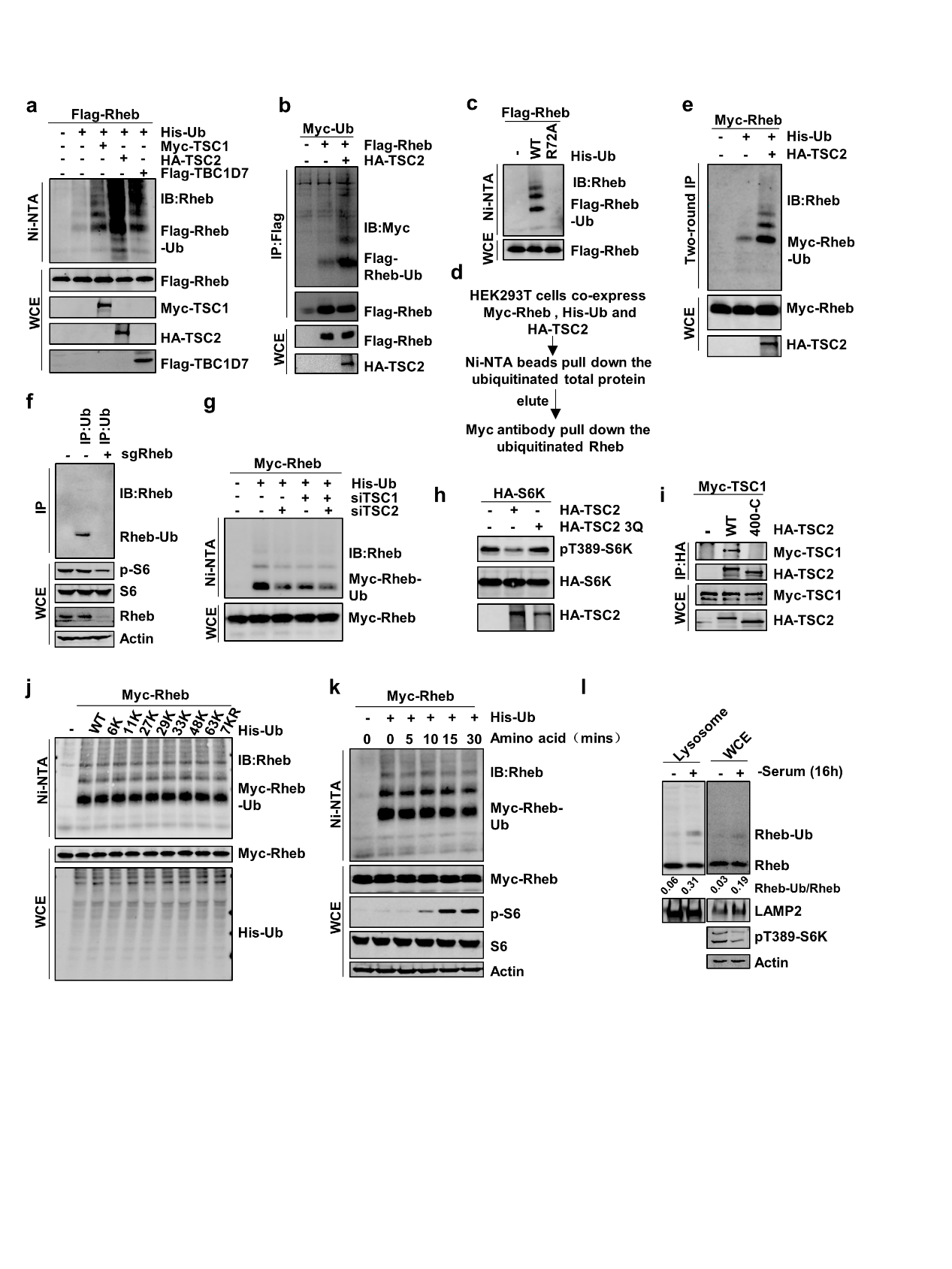
**

**Fig. S1 TSC complex and EGF signaling promote the ubiquitination of Rheb.** (a). Flag-Rheb, His-Ub and TSC complexes were co-transfected in HEK293 cells to detect the ubiquitination of Rheb. Rheb ubiquitination was analyzed as shown in Fig. 1a. (b). The ubiquitination of Rheb was detected by immunoprecipitates (IP) assay, and IP was performed from HEK293T cells transfected with Myc-Ub, Flag-Rheb and HA-TSC2. (c). Flag-Rheb was co-transfected with Ub-WT or Ub-R72A mutant to detect Rheb ubiquitination level. Rheb ubiquitination was analyzed as shown in Fig. 1a. (d). Experimental procedure for detecting Rheb ubiquitination by two rounds of IP. (e). TSC2 and His-Ub were co-transfected and the ubiquitination of Rheb was detected by two rounds of IP assay. (f). Endogenous Rheb ubiquitination was detected in both control and Rheb-deficient cells. (g). Myc-Rheb, His-Ub and siRNA targeting TSC1 orTSC2 were transfected into HEK293T cells, respectively. Rheb ubiquitination was analyzed as shown in Fig. 1a. (h). Co-transfection of HA-S6K, HA-TSC2-3Q (K1595Q, K1596Q and R1597Q) or HA-TSC2 in HEK293T. mTORC1 activity was analyzed by immunoblotting. (i). The interaction between TSC1 and TSC2-WT or TSC2 mutant (TSC2 400-C) was detected by Co-IP assay. (j). HEK293T cells were co-transfected with Myc-Rheb and Ub-WT or K only mutants (all the lysine in ubiquitin were mutated to arginine except for the indicated lysine). Rheb ubiquitination was analyzed as shown in Fig. 1a. (k). Rheb ubiquitination was detected under amino acid stimulation. (l). Lysosome was purified from cells with or without serum starvation for 24 hours. The levels of ubiquitinated Rheb were examined in lysosome samples.
