# Supplementary material for: Ubiquitination of Rheb governs growth factor-induced mTORC1 activation
Source: Cell Res. 2018 Dec 4;29(2):136–50. doi: 10.1038/s41422-018-0120-9 (PMC6355928; doi:10.1038/s41422-018-0120-9)
Supplement: Supplementary file 2 — Supplementary information, Fig. S2 [file 41422_2018_120_MOESM2_ESM.docx]

**Supplementary information, Fig. S2**

**
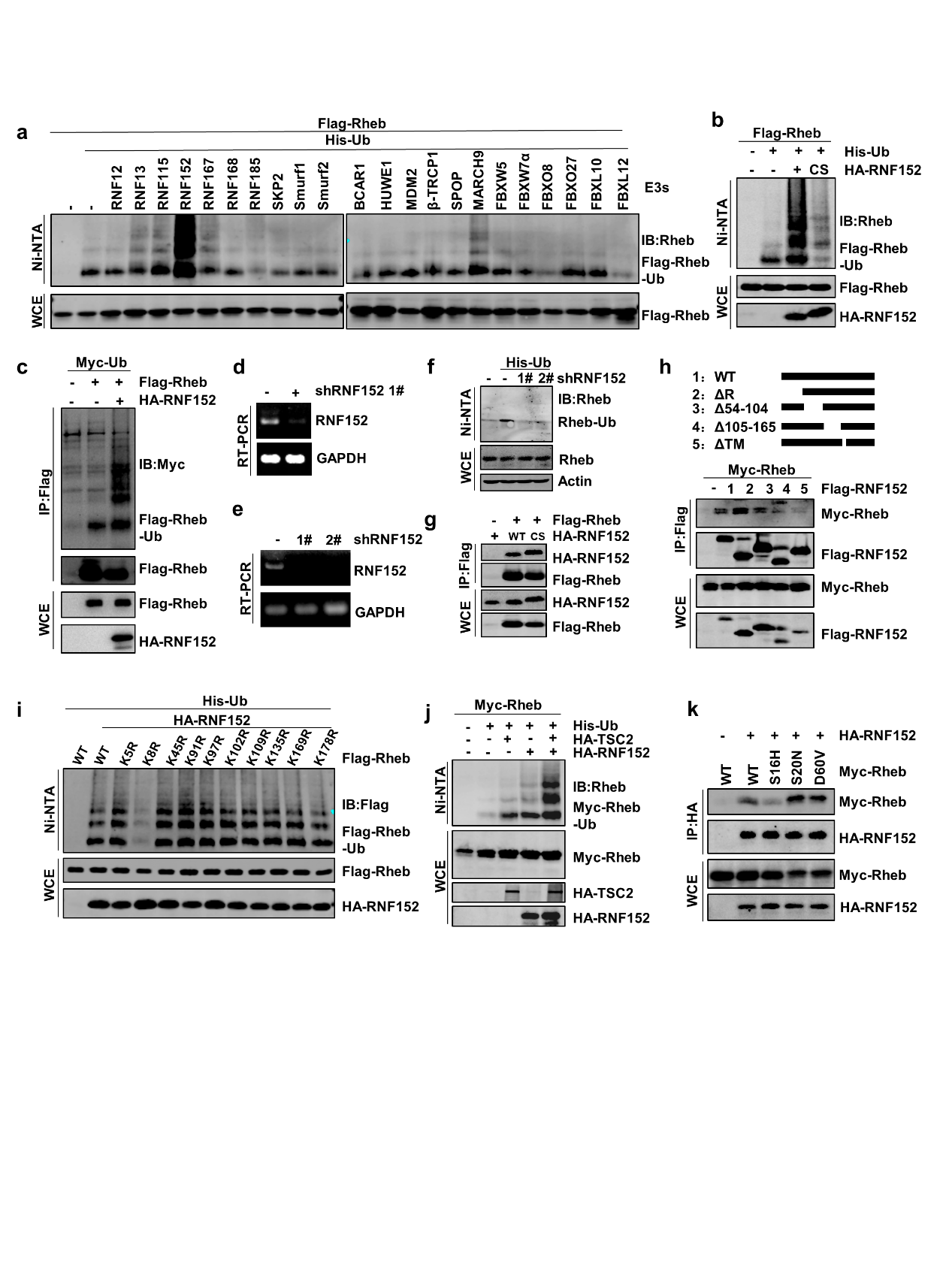
**

**Fig. S2. RNF152 is involved in TSC2-mediated Rheb ubiquitination.** (a). Screening for the E3 ubiquitin ligase targeting Rheb. Flag-Rheb, His-Ub were co-transfected in HEK293T cells with the indicated E3 ligases, respectively, and the ubiquitination experiment was performed via Ni-NTA. (b). His-Ub, Flag-Rheb, HA-RNF152 or RNF152-CS were co-expressed in HEK293T cells as indicated and the ubiquitination levels were detected via Ni-NTA. (c). The ubiquitination of Rheb by RNF152 was analyzed by IP assay. (d and e). Knockdown efficiency of RNF152 was detected in HEK293T (d) and H1299 cells (e) via PT-PCR. (f). The ubiquitination of endogenous Rheb was detected in RNF152 knockdown H1299 cells. The knockdown efficiency of RNF152 was detected by RT-PCR in Supplementary information, Fig. S2e. (g). The binding of Rheb to RNF152-WT or RNF152-CS was analyzed by co-IP assay in HEK293T cells. (h). Different fragments of RNF152 and Myc-Rheb were co-expressed in HEK293T cells. The interaction between Rheb and RNF152 mutants was detected by Co-IP assay. (i). The ubiquitination of lysine mutated Rheb mutant was analyzed by co-expressing His-Ub, HA-RNF152 together with Rheb or Rheb mutants in HEK293T cells. (j). TSC2 promoted the RNF152-induced Rheb ubiquitination. His-Ub, Myc-Rheb, HA-RNF152 and HA-TSC2 were co-expressed in HEK293T cells. Ubiquitination assay was performed via Ni-NTA. (k). The binding of RNF152 to different Rheb mutants (inactive form: S20N and D60V, active form: S16H) was detected in HEK293T cells.
