# Supplementary material for: Ubiquitination of Rheb governs growth factor-induced mTORC1 activation
Source: Cell Res. 2018 Dec 4;29(2):136–50. doi: 10.1038/s41422-018-0120-9 (PMC6355928; doi:10.1038/s41422-018-0120-9)
Supplement: Supplementary file 3 — Supplementary information, Fig. S3 [file 41422_2018_120_MOESM3_ESM.docx]

**Supplementary information, Fig. S3**


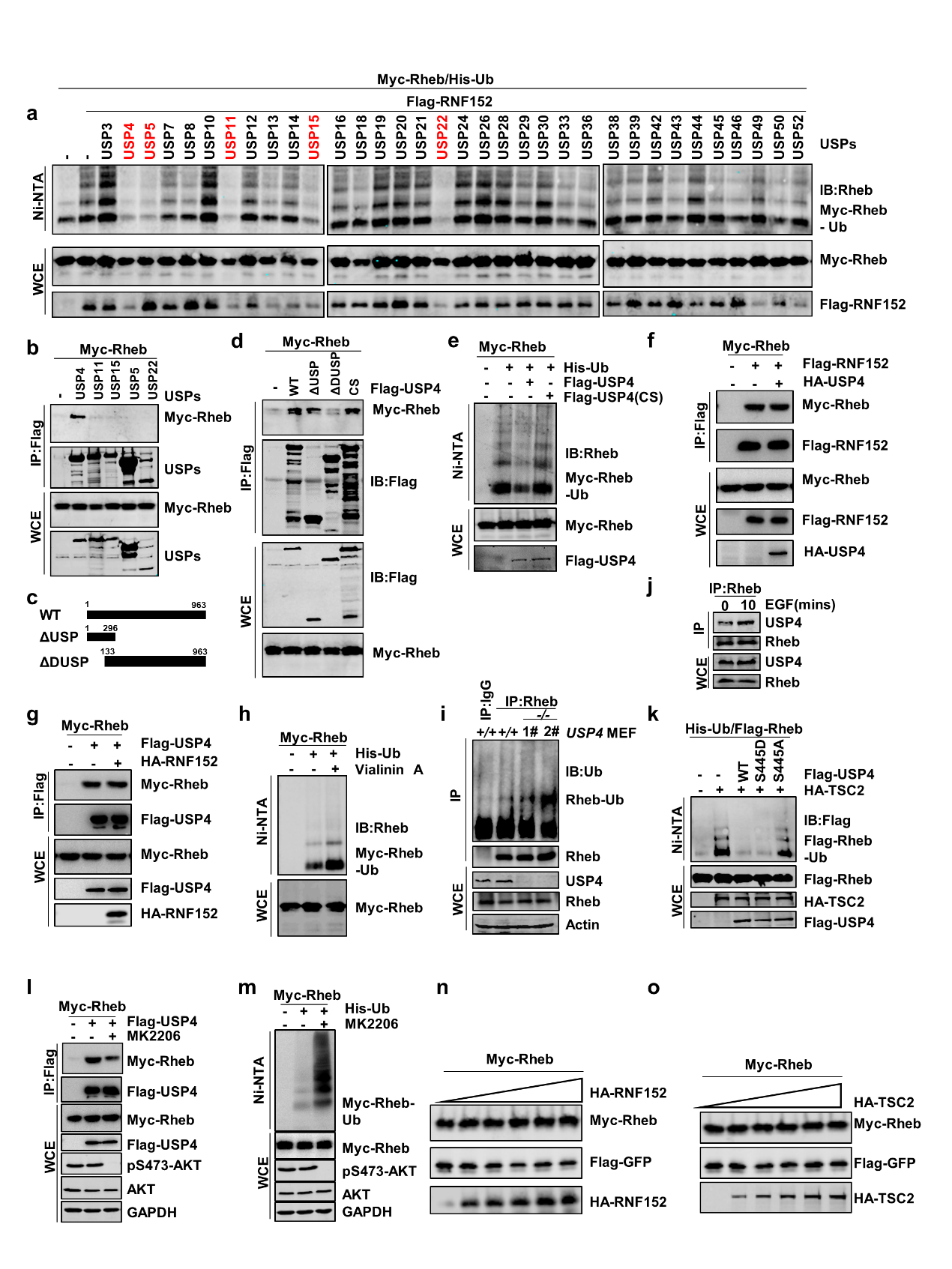


**
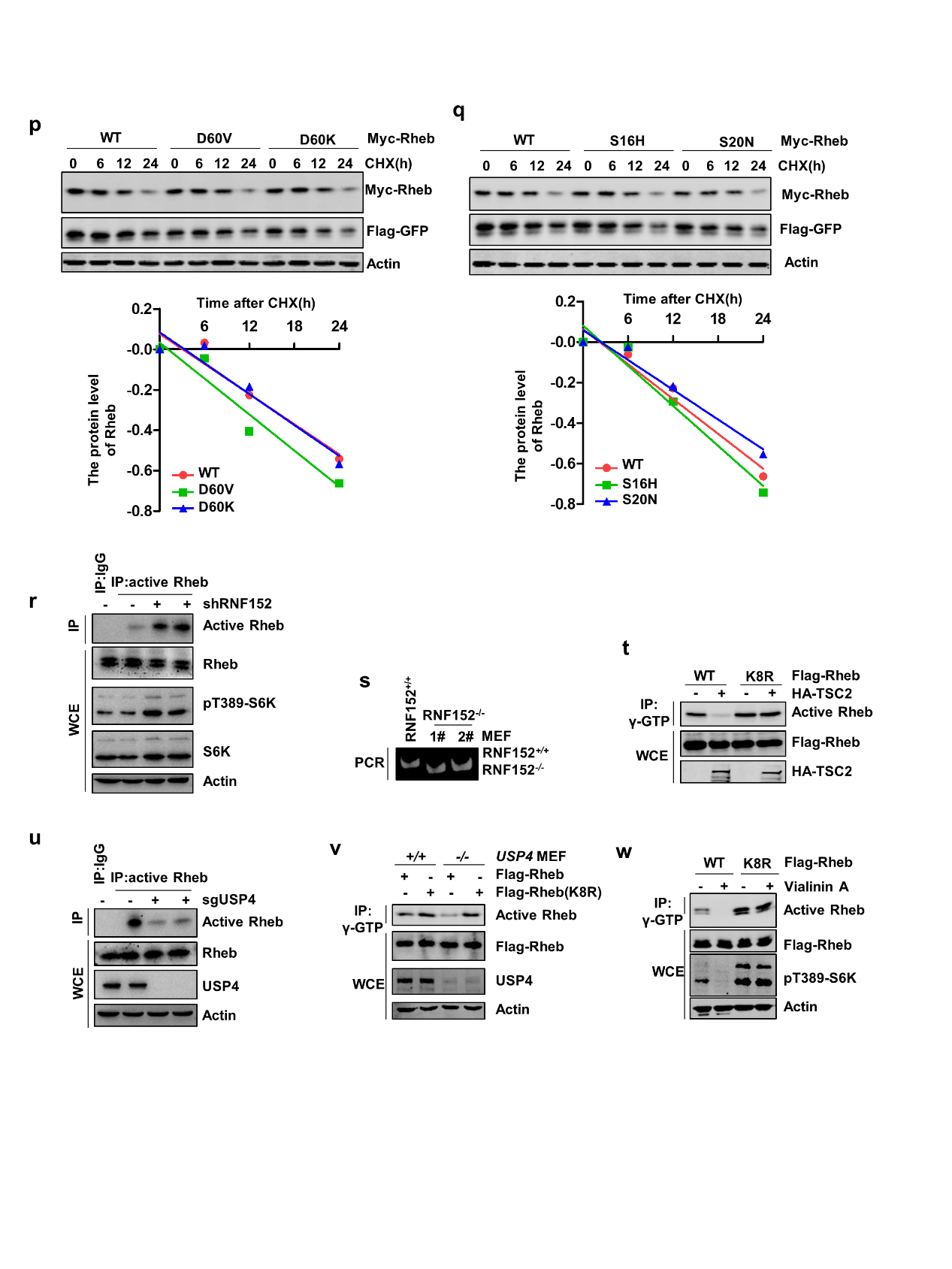
**

**Fig. S3 USP4 promotes Rheb activation through removing the ubiquitin from Rheb.** (a). The specific DUB of Rheb was screened. Myc-Rheb, His-Ub and the indicated DUBs were co-expressed in HEK293T cells. The ubiquitination was analyzed by Ni-NTA. (b). The interaction between Myc-Rheb and USPs was analyzed by Co-IP assay in HEK293T cells. (c and d). The binding of Myc-Rheb to different USP4 mutants (DUSP, USP, CS) (c) was analyzed by Co-IP assay (d) in HEK293T cells. (e). The ubiquitination of Rheb was detected by co-expressing Myc-Rheb, His-Ub, USP4-WT or USP4-CS in HEK293T cells. (f and g). Rheb and the indicated plasmids were co-expressed in HEK293T cells and protein interaction was analyzed by co-IP assay. (h). Rheb ubiquitination was detected with treatment of Vialinin A (2 μm, 4 h) in HEK293T cells. (i). The ubiquitination of endogenous Rheb was detected in USP4 -/- and USP4+/+ MEF cells. (j). The interaction between Rheb and USP4 under EGF stimulation. (k). Regulation of TSC2-mediated Rheb ubiquitination by different USP4 mutants (WT, S445A and S445D). (l). The interaction between Rheb and USP4 was detected with the treatment of the AKT inhibitor MK2206 (2 μm, 4 hours). (m). The Rheb ubiquitination levels were detected with the treatment of AKT inhibitor MK2206 for 4 hours. (n and o). Rheb and RNF152(n) or TSC2(o) were co-expressed in HEK293T cells. Protein levels of Rheb and RNF152/TSC2 were analyzed by Western Blotting. (p and q). Rheb or mutants was expressed in HEK293T cells. The protein levels of Rheb was analyzed by WB with the treatment of CHX (10 μg/ml) for the indicated time and the Rheb protein abundance was quantified by ImageJ. (r). The activity of endogenous Rheb was detected in WT or RNF152 knockdown H1299 cells, which were cultured in normal medium. The knockdown efficiency of RNF152 was detected via RT-PCR in Supplementary information, Fig. S2e. (s). Identification of RNF152-KO MEF cells by genomic DNA PCR. (t). The effect of TSC2 on the activity of Rheb-WT and Rheb-K8R was examined in HEK293T cells, which were cultured in normal medium. (u). Rheb activity was measured in WT and USP4-KO cell lines, which were cultured in normal medium. (v). Rheb-WT and K8R were transfected in MEF cells and the activity of Rheb was detected. (w). The transfected cells were treated with USP4 inhibitor Vialinin A (2 μm) and the activity of Rheb-WT and K8R mutants was analyzed.
