# Supplementary material for: Ubiquitination of Rheb governs growth factor-induced mTORC1 activation
Source: Cell Res. 2018 Dec 4;29(2):136–50. doi: 10.1038/s41422-018-0120-9 (PMC6355928; doi:10.1038/s41422-018-0120-9)
Supplement: Supplementary file 4 — Supplementary information, Fig. S4 [file 41422_2018_120_MOESM4_ESM.docx]

**Supplementary information, Fig. S4**

**
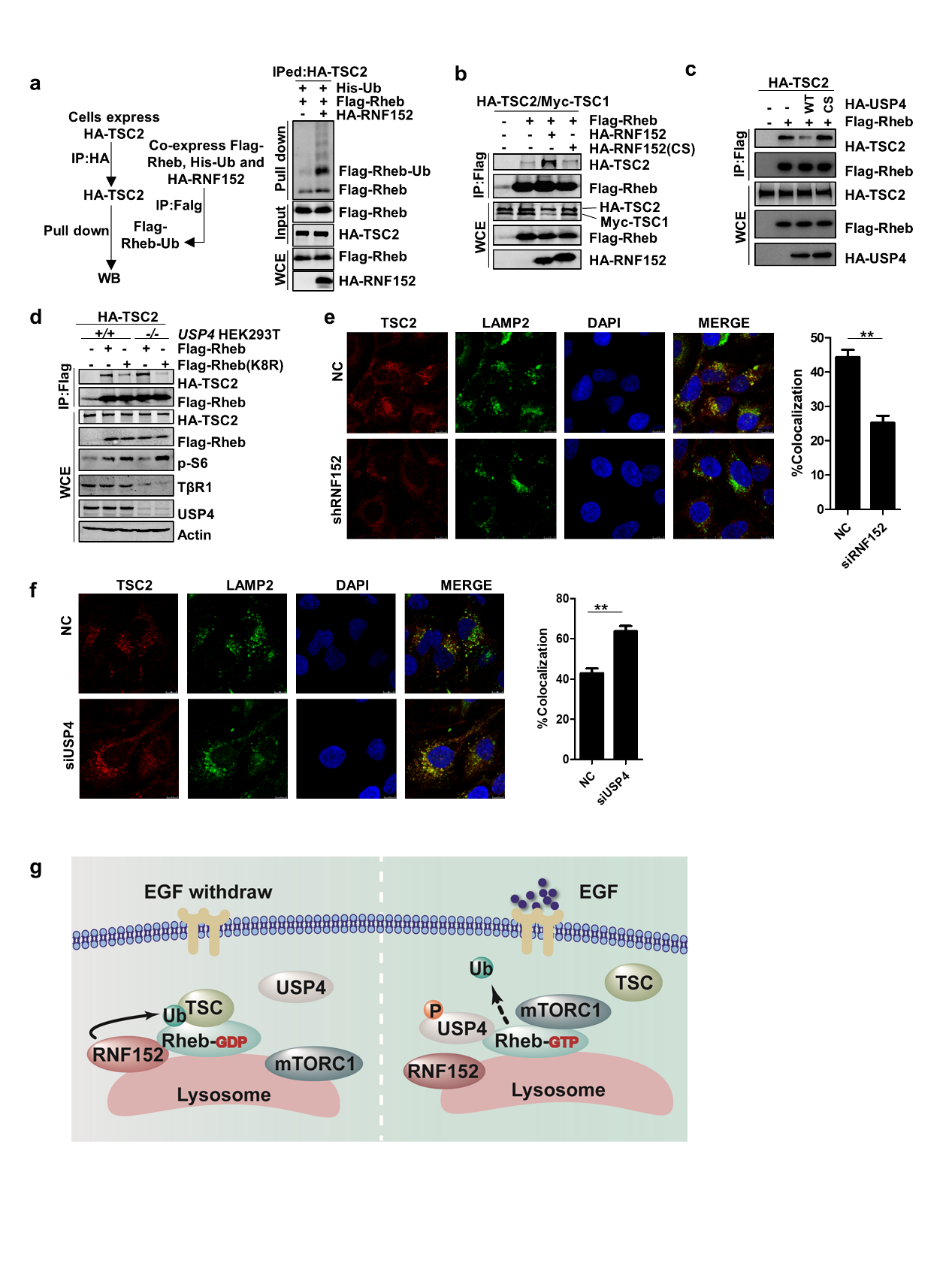
**

**Fig. S4 Ubiquitinated Rheb promotes its interaction with TSC.** (a). The binding of TSC2 to WT-Rheb or the ubiquitinated Rheb was examined in vitro. The schematic is shown on the left. (b). Flag-Rheb, Myc-TSC1, HA-TSC2, RNF152-WT or RNF152-CS were co-expressed in HEK293T cells and the interaction between Flag-Rheb and HA-TSC2 was detected by Co-IP assay. (c). The interaction between Flag-Rheb and HA-TSC2 was detected by expressing USP4-WT or USP4-CS in HEK293T cells. (d). The binding of TSC2 to Rheb-WT or Rheb-K8R mutations were examined in USP4-WT or USP4-KO cells. (e and f). The effects of RNF152(e) or USP4(f) on the colocalization of TSC2 and LAMP2 were detected In HEK293T cells by immunofluorescence. The quantified data of colocalization were present as mean ± SEM (right), p value was considered statistically significant, ∗∗ denote p values of < 0.01. Data was analyzed by student’s t test. (g). Model for the regulation of Rheb ubiquitination by EGF signal: RNF152-mediated ubiquitination of Rheb promotes the interaction between Rheb and TSC2 and thereby inhibiting Rheb activation under the condition of growth factor deficiency. Moreover, the RNF152 mediated Rheb ubiquitination depends on the inactivation of Rheb by TSC2, which forms a positive feedback loop in controlling Rheb activity. USP4 plays an opposite role in response to growth factor by deubiquitination of Rheb and ultimately activates Rheb.
