# Supplementary material for: Ubiquitination of Rheb governs growth factor-induced mTORC1 activation
Source: Cell Res. 2018 Dec 4;29(2):136–50. doi: 10.1038/s41422-018-0120-9 (PMC6355928; doi:10.1038/s41422-018-0120-9)
Supplement: Supplementary file 5 — Supplementary information, Fig. S5 [file 41422_2018_120_MOESM5_ESM.docx]

**Supplementary information, Fig. S5**

**
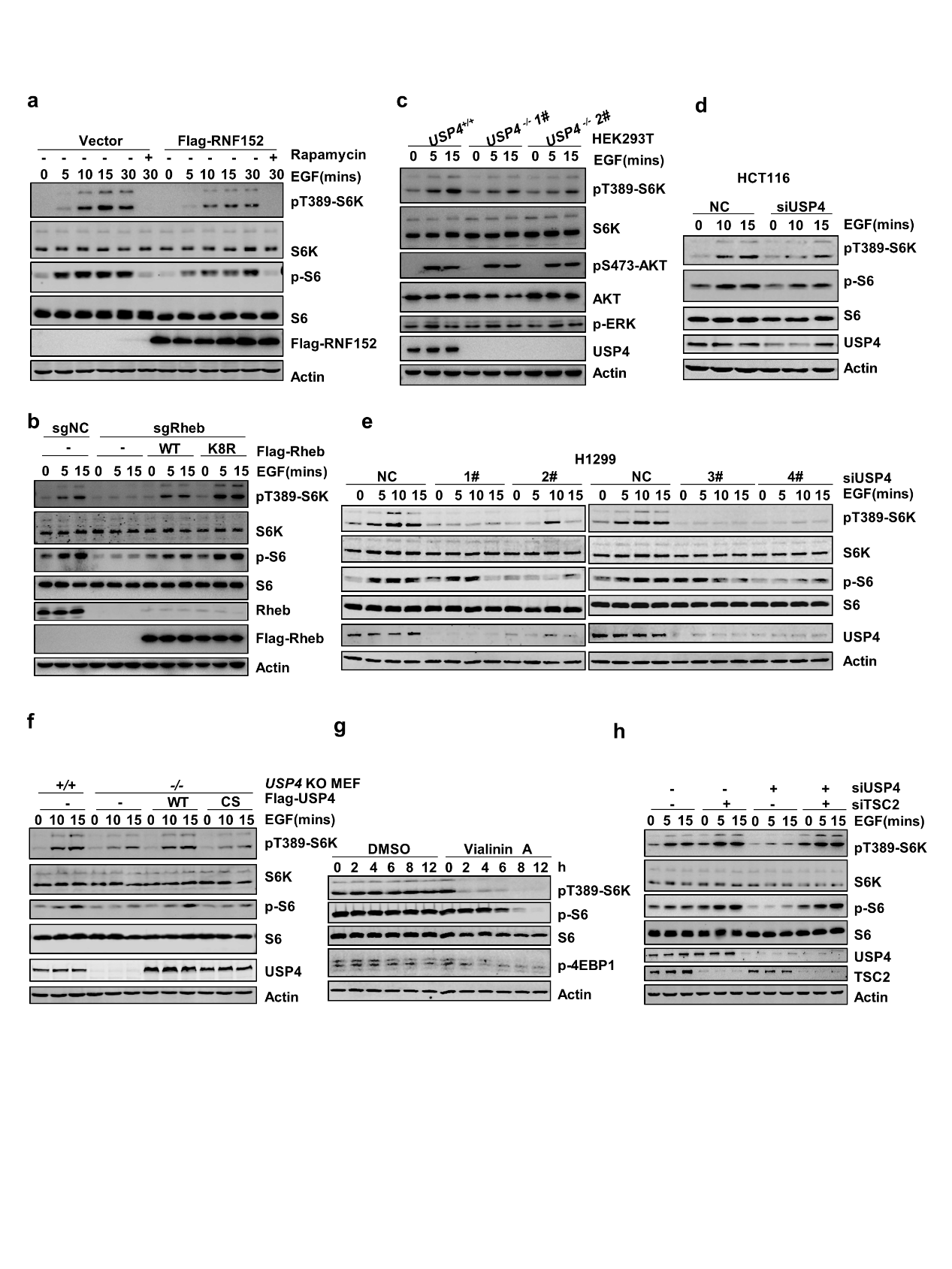
**

**Fig. S5. RNF152 and USP4 regulate mTORC1 activation.** (a). Overexpressing RNF152 inhibited the EGF induced mTORC1 activation. (b). Rheb-WT or Rheb-K8R was overexpressed in the indicated cells and activation of mTORC1 was detected. (c). The mTORC1 activity was detected in indicated cells. (d and e). USP4 knockdown reduced the EGF-induced mTORC1 activation in HCT116 cells(d) and H1299 (e) cells. (f). EGF-induced activation of mTORC1 was detected in MEFs expressing USP4-WT or USP4-CS. (g). The effect of Vialinin A (2 μM) treatment on the mTORC1 activation. (h). The mTORC1 activation was analyzed in USP4 and TSC2 knockdown cells.
