# Supplementary material for: Ubiquitination of Rheb governs growth factor-induced mTORC1 activation
Source: Cell Res. 2018 Dec 4;29(2):136–50. doi: 10.1038/s41422-018-0120-9 (PMC6355928; doi:10.1038/s41422-018-0120-9)
Supplement: Supplementary file 6 — Supplementary information, Fig. S6 [file 41422_2018_120_MOESM6_ESM.docx]

**Supplementary information, Fig. S6**

**
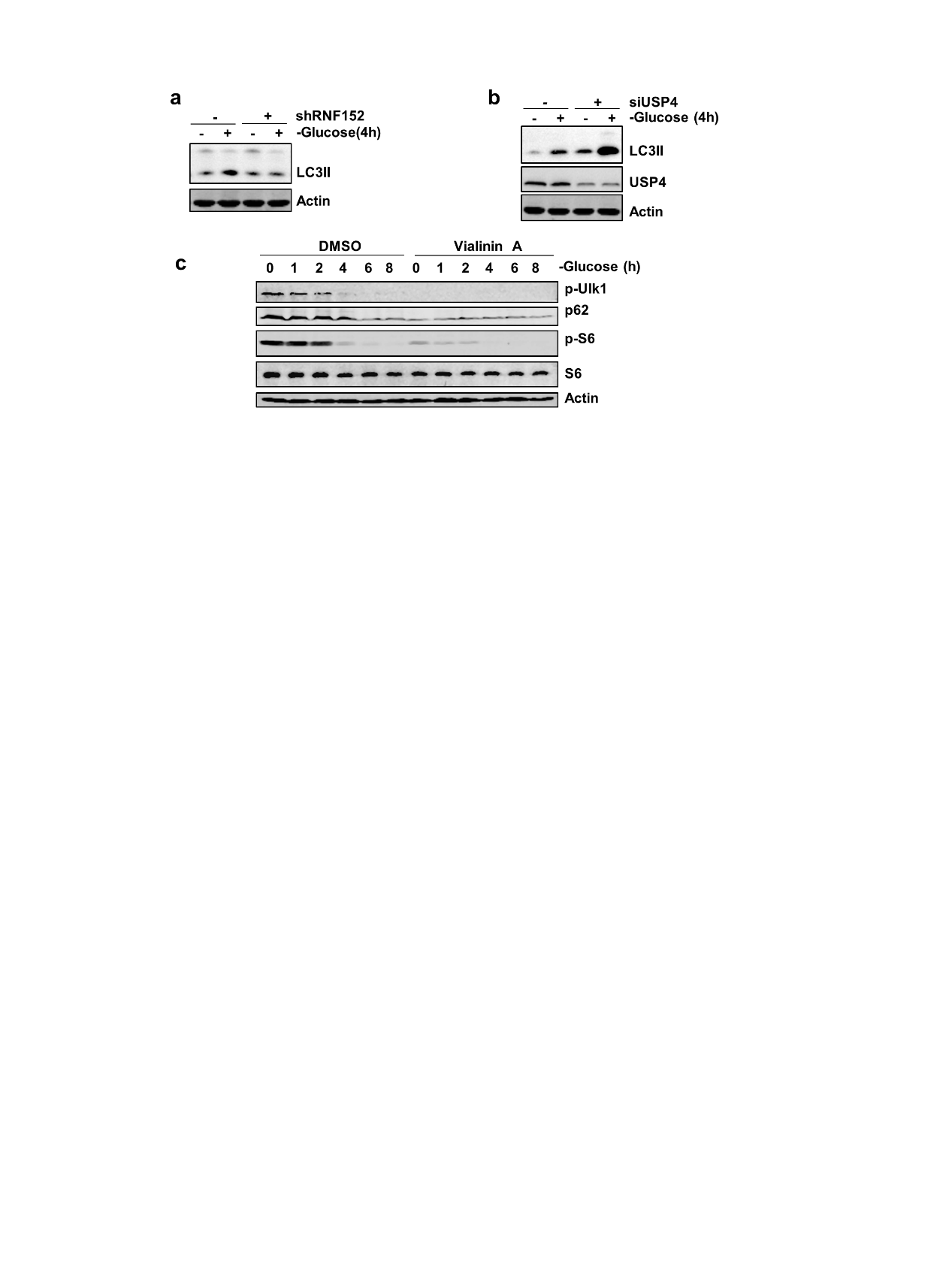
**

**Fig. S6 Rheb ubiquitination regulates cell autophagy, cell proliferation and cell size.** (a). The protein levels of LC3II was analyzed in HEK293T cells after glucose starvation. Cells were harvested with the treatment of Bafilomycin A1(100 nM) treatment for 4 hours. The knockdown efficiency of RNF152 was detected by RT-PCR in Supplementary information, Fig. S2d. (b). The protein levels of LC3II were analyzed in HEK293T cells after glucose starvation. Cells were treated with Bafilomycin A1(100 nM) for 4 hours before being harvested. (c). Protein levels of p-ULK1 and p62 were analyzed with or without Vialinin A (2 μM) treatment (8 hours) under glucose starvation for the indicated time in HEK293T cells.
