# Supplementary material for: Ubiquitination of Rheb governs growth factor-induced mTORC1 activation
Source: Cell Res. 2018 Dec 4;29(2):136–50. doi: 10.1038/s41422-018-0120-9 (PMC6355928; doi:10.1038/s41422-018-0120-9)
Supplement: Supplementary file 7 — Supplementary information, Fig. S7 [file 41422_2018_120_MOESM7_ESM.docx]

**Supplementary information, Fig. S7**

**
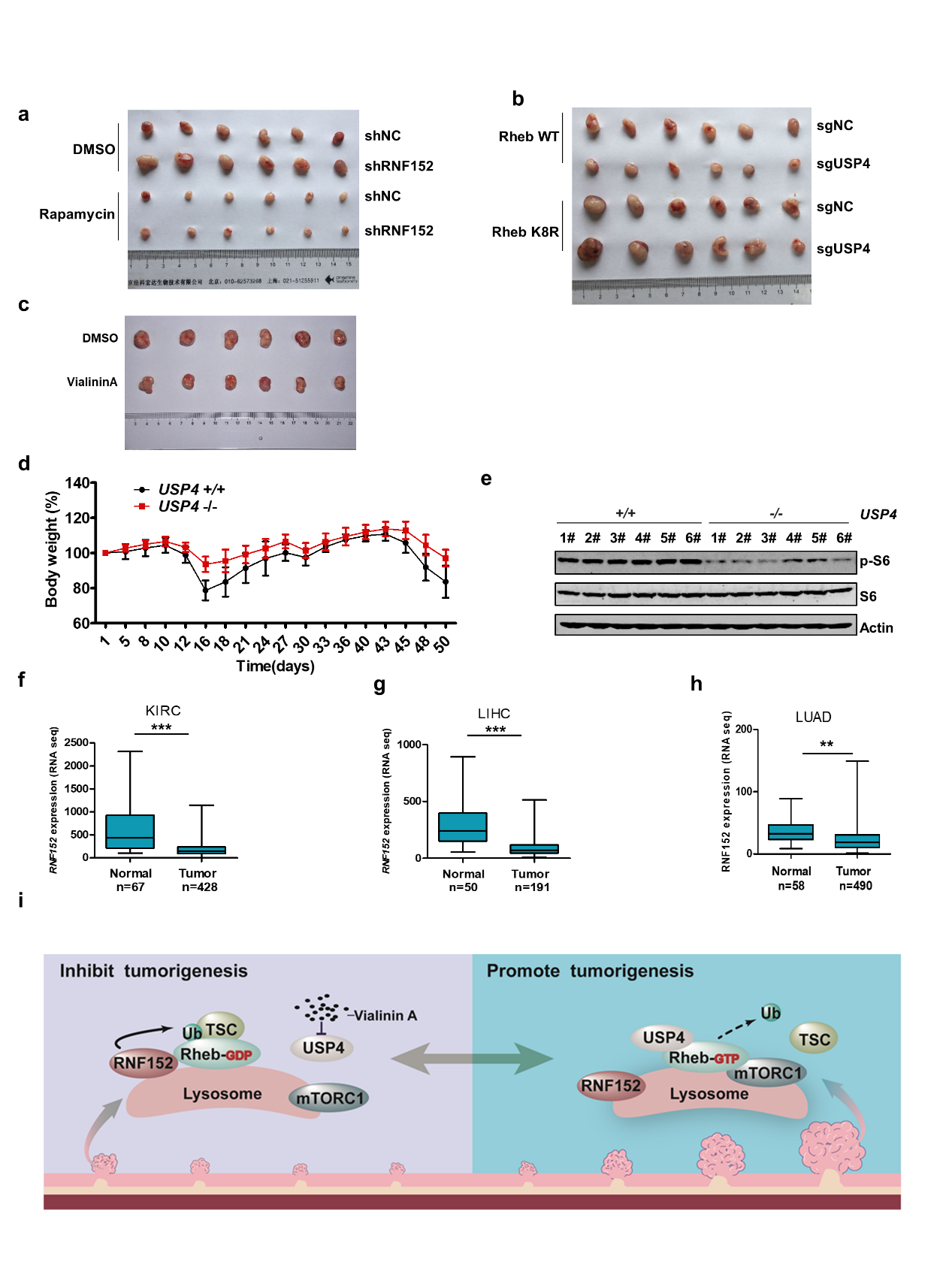
**

**Fig. S7 Regulation of tumor growth by RNF152 or USP4 in an mTOR-dependent manner.** (a). WT or RNF152 deficient SW620 cells were injected into nude mice subcutaneous, respectively. After injection for 6 days, mice were injected Rapamycin for 5 consecutive days through intraperitoneal injection. (b). WT or USP4 deficient SW620 cells stably expressing Rheb-WT or Rheb-K8R were injected into nude mice subcutaneous. (c). HCT116 cells were injected into nude mice subcutaneous, followed by Vialinin A treatment. (d). Percentage change in body weight of USP4*^-/-^* and USP4*^+/+^* mice during azoxymethane and DSS treatment. Azoxymethane was injected at day 1. (e). mTORC1 activity was analyzed in tumor samples from USP4*^-/-^* and USP4*^+/+^* colorectal mice. (f, g and h). The expression levels of RNF152 in various cancers based on the TCGA database, p value was considered statistically significant, ∗∗ and ∗∗∗ denote p values of < 0.01 and 0.001, respectively, data were analyzed by student’s t test. (i). Model of ubiquitination of Rheb on tumorigenesis. With the treatment of Vialinin A treatment, the activation of USP4 is inhibited, RNF152 induces the ubiquitination of Rheb specifically. The ubiquitination and inactivation of Rheb forms a positive feedback loop in controlling Rheb activity and inhibits tumorigenesis effectively. While in turn, USP4, acts as a counterpart of RNF152, plays a key role in mTORC1 signaling pathway, removes ubiquitin moiety from Rheb, leads to releases of TSC2 from Rheb and reactivates Rheb, and then promotes mTORC1 activation and tumorigenesis.
